# Alternative methods improve the accuracy of genomic prediction using information from a causal point mutation in a dairy sheep model

**DOI:** 10.1186/s12864-019-6068-4

**Published:** 2019-09-18

**Authors:** Claire Oget, Marc Teissier, Jean-Michel Astruc, Gwenola Tosser-Klopp, Rachel Rupp

**Affiliations:** 1 GenPhySE, Université de Toulouse, INRA, ENVT, Castanet-Tolosan, France; 2 Institut de l’Elevage, 31321 Castanet-Tolosan, France

**Keywords:** Genomics, Genomic evaluation, Genome-wide association study, Dairy sheep, Causal mutation

## Abstract

**Background:**

Genomic evaluation is usually based on a set of markers assumed to be linked with causal mutations. Selection and precise management of major genes and the remaining polygenic component might be improved by including causal polymorphisms in the evaluation models. In this study, various methods involving a known mutation were used to estimate prediction accuracy. The *SOCS2* gene, which influences body growth, milk production and somatic cell scores, a proxy for mastitis, was studied as an example in dairy sheep.

**Methods:**

The data comprised 1,503,148 phenotypes and 9844 54K SNPs genotypes. The *SOCS2* SNP was genotyped for 4297 animals and imputed in the above 9844 animals. Breeding values and their accuracies were estimated for each of nine traits by using single-step approaches. Pedigree-based BLUP, single-step genomic BLUP (ssGBLUP) involving the 54K ovine SNPs chip, and four weighted ssGBLUP (WssGBLUP) methods were compared. In WssGBLUP methods, weights are assigned to SNPs depending on their effect on the trait. The ssGBLUP and WssGBLUP methods were again tested after including the *SOCS2* causal mutation as a SNP. Finally, the Gene Content approach was tested, which uses a multiple-trait model that considers the *SOCS2* genotype as a trait.

**Results:**

EBV accuracies were increased by 14.03% between the pedigree-based BLUP and ssGBLUP methods and by 3.99% between ssGBLUP and WssGBLUP. Adding the *SOCS2* SNP to ssGBLUP methods led to an average gain of 0.26%. Construction of the kinship matrix and estimation of breeding values was generally improved by placing emphasis on SNPs in regions with a strong effect on traits. In the absence of chip data, the Gene Content method, compared to pedigree-based BLUP, efficiently accounted for partial genotyping information on *SOCS2* as accuracy was increased by 6.25%. This method also allowed dissociation of the genetic component due to the major gene from the remaining polygenic component.

**Conclusions:**

Causal mutations with a moderate to strong effect can be captured with conventional SNP chips by applying appropriate genomic evaluation methods. The Gene Content method provides an efficient way to account for causal mutations in populations lacking genome-wide genotyping.

**Electronic supplementary material:**

The online version of this article (10.1186/s12864-019-6068-4) contains supplementary material, which is available to authorized users.

## Background

By estimating genetic parameters, such as the heritability of a given trait, individuals could be selected according to their genetic value, based on the Estimated Breeding Values (EBVs) for that trait, and the whole species might be genetically improved for traits such as production, health, morphology, etc. Schemes were therefore set up to select improved males and use their semen on livestock breeding farms. The genetic architecture of traits of interest in livestock species has been widely studied since the 1920s [1]. Such traits can be governed by genes with small effects but also by large Quantitative Trait *Loci* (QTLs) or major genes. Studies of this complex architecture have been facilitated by new technologies and molecular markers such as microsatellites or single nucleotide polymorphisms (SNPs) which make it possible to detect the regions of the genome responsible for genetic variation and measure their respective effects [1].

Genetic selection methods were initially based on pedigree approaches and the method first employed to estimate breeding values was the Best Linear Unbiased Prediction method (BLUP) [2]. Approaches based on SNP chips were then quickly developed from the 1990s onwards [3–6]. These approaches allowed EBVs to be estimated from pedigree information, from genotyping data about a proportion of the population (males in testing stations for example), and from performance data. Performance data could be based on means of progeny performance, e.g. Daughter Yield Deviations (DYD), as in the two-step pedigree-based BLUP [2] or Genomic BLUP (GBLUP) [3, 4] approaches. More recently, methods to directly use raw phenotypes of non-genotyped individuals in the so-called single-step GBLUP approach (ssGBLUP) were developed [5, 6]. A few studies showed that the prediction accuracy of evaluations could be increased by using ssGBLUP, rather than two-step pedigree-based BLUP or GBLUP approaches [7–9].

Two studies, in the same dairy Lacaune breed sheep population investigated here, resulted in the development of genetic evaluation models based on molecular markers [10, 11]. Duchemin et al. (2012) [10], after comparing the BLUP, Bayes Cπ, Partial Least Squares (PLS), and sparse PLS methods, reported that depending on the trait and compared to the BLUP method, EBV accuracies could be increased by 18 to 25% by including markers in the models, with minor differences between the genomic approaches. Baloche et al. (2014) [11] then adopted BLUP-like methods to implement a single-step model in the evaluation and compared pseudo-BLUP and pseudo-ssGBLUP (using all rams and their DYDs in both methods), and regular ssGBLUP (using individual phenotypes and pedigree in an animal model), and obtained the best results with regular ssGBLUP. In 2015, the ssGBLUP approach was therefore implemented in the French official genetic evaluations of Lacaune sheep [12] and is used as a reference method in this study. However, the previously tested methods, and the one currently used in official evaluations, do not allow a higher weight to be assigned to markers in QTL regions or to a major gene such as the *SOCS2* gene, which influences many traits due to the mutation present in this population.

In the ssGBLUP approach, all SNPs are given the same weight during construction of the relationship matrix. Methods have since been developed to assign more weight to markers that are more strongly associated with the trait under study [13, 14] or to a major gene influencing the trait, in a multi-trait approach (called Gene Content) [15]. These methods have been tested in goats and have been shown to improve evaluation accuracy [16–18].

In this study, we chose the causal mutation for mastitis resistance characterized in a dairy sheep association study: namely the R96C point mutation in the *SOCS2* gene (suppressor of cytokine signaling 2) [19]. This mutation, which consists of a modification in a single base pair (substitution of an allelic base C into an allelic base T), introduces a SNP at this *locus* and modifies the affinity of the protein for its ligand. Rupp et al. (2015) [19] showed that this mutation, with a Minor Allele Frequency (MAF) of 21.7% in the population (468 rams from testing stations), was strongly associated (deteriorated health) with the Somatic Cell Count (SCC) trait, considered as a proxy for mastitis (i.e. it explained 12% of the genetic variance of this trait), but was also favourably associated with size, weight and, to a lesser extent, with milk yield traits. This pleiotropic gene therefore seemed a good candidate for testing several approaches to exploit information about QTL or causal mutations in evaluation models.

Thus the objectives of this study were: (i) to test evaluation methods that allowed inclusion of information about a causal mutation, using the example of the dairy sheep *SOCS2* gene point mutation, and (ii) to analyze the effect of applying different methods to utilize this additional information on prediction accuracies and on EBV trends over time.

## Results

### Imputation of *SOCS2* genotypes

**Fig. 1:**
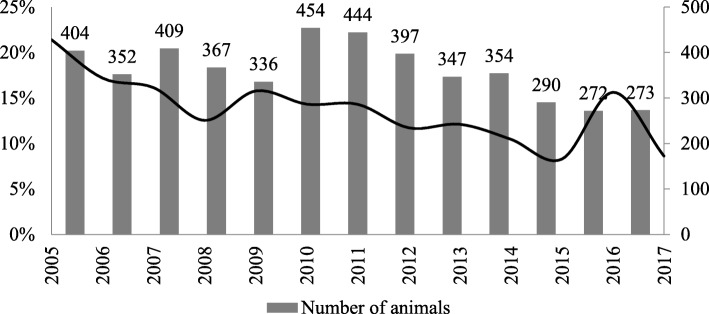
Evolution of the frequency of the *SOCS2* T allele, associated with increased susceptibility to mastitis in 4699 rams used for artificial insemination

We obtained a Concordance Rate (CR) of 0.988 for imputation of the *SOCS2* genotypes, i.e. 33 imputation errors among the 1432 individuals in the imputation validation population. The result of this imputation provided us with the *SOCS2* genotypes for the entire genotyped population, with a MAF of 0.14 for the mutated T allele associated with higher susceptibility to mastitis. The MAF trend in the population of 4699 AI rams is shown in Fig. 1. The MAF decreased from 0.21 in 2005 to 0.09 in 2017.

### Linkage Disequilibrium (LD)

The map coverage of the chip attained 2444 Mb and the mean SNP interval was 0.064 Mb. The mean *r*^2^ were 0.25, 0.16, 0.12, 0.09, and 0.08 for a distance between pairs of SNPs of < 0.02 Mb, [0.02–0.04 Mb], [0.04–0.06 Mb], [0.06–0.08 Mb], and [0.08–0.10 Mb], respectively, and < 0.08 for all the other distance categories. A visualization of *r*^2^ according to distance between SNP is provided in Additional file 1: Figure S1.

The *r*^2^ measure of LD between the 40 markers closest to *SOCS2* is represented in Additional file 1: Figure S2. The LD of *SOCS2* with the other SNP markers ranged from zero to 0.47 with OAR3_138135461.1, which was 0.229 Mb pairs away from *SOCS2*. The average LD between the *SOCS2* SNP and the 10 previous SNPs on the chip was 0.17. The category containing the distance between the *SOCS2* SNP and the SNP most linked to the *SOCS2* SNP (0.229 Mb) was the interval [0.22, 0.24], for which we obtained a mean *r*^2^ of 0.049 on the whole chip. The *SOCS2* SNP mutation was therefore in strong LD with some of the other SNPs in the region.

### Genetic parameters

The (co)variance parameters estimated and used in this study are presented in Additional file 1: Figure S3. The variance estimates for the single-trait models were very similar, whether estimated from pedigree or genomic relationships. Heritabilities were 0.50 and 0.61–0.62 for FC and PC, respectively. For Milk Yield (MY), Fat Yield (FY) and Protein Yield (P)Y, they were 0.37, 0.37, and 0.39, respectively. For Teat Angle (TA), Udder Cleft (UC) and Udder Depth (UD), they were 0.39, 0.34, 0.27–0.28 respectively, and for Lactation Somatic Cell Score (LSCS) 0.17–0.18. Similar results were obtained using the two-trait models (Additional file 1: Figure S3).

Genetic correlations between the *SOCS2* gene content trait and the other traits, and the genetic variances explained by the *SOCS2* gene using the pedigree-based Gene Content method are presented in Table 1. The absolute values of the genetic correlations ranged from 0.02 (TA) to 0.34 (LSCS). The six traits most correlated with the *SOCS2* gene content trait were: LSCS (***r***_***g***_ = 0.34), PY (***r***_***g***_ = 0.29), MY (***r***_***g***_ = 0.25), UD (***r***_***g***_ = − 0.19), FY (***r***_***g***_ = 0.18) and FC (***r***_***g***_ = − 0.14). This was confirmed by the genetic variances explained by the *SOCS2* gene that ranged from 0.05% (TA) to 11.24% (LSCS).

**Table 1:** Genetic correlations between the *SOCS2* gene content trait and the traits of interest (r_g_) and genetic variances (σ_g_^2^) explained by the *SOCS2* gene obtained using the pedigree-based Gene Content method

Trait	r_g_ with *SOCS2*	σ_g_^2^ explained by *SOCS2*
MY	0.25	6.18%
FY	0.18	3.22%
PY	0.29	8.55%
FC	–0.14	1.88%
PC	–0.06	0.41%
LSCS	0.34	11.24%
TA	–0.02	0.05%
UC	–0.07	0.56%
UD	–0.19	3.71%

*Abbreviations*: *MY* Milk Yield, *FY* Fat Yield, *PY* Protein Yield, *FC* Fat Content, *PC* Protein Content, *LSCS* Somatic Cell Score, *TA* Teat Angle, *UC* Udder Cleft, *UD* Udder Depth

### GBLUP and WssGBLUP methods improve prediction accuracies

The prediction accuracies and gains obtained with the different evaluation methods and traits are shown in Table 2. Prediction accuracies ranged from 0.498 to 0.561 for MY, from 0.330 to 0.486 for FY and PY, and from 0.684 to 0.762 for FC and PC. They ranged from 0.421 to 0.471 for LSCS, and from 0.336 to 0.538 for udder type traits.

**Table 2:** Prediction accuracies of different genetic evaluation methods for each trait using information about the *SOCS2* gene or not

		Trait								
		MY	FY	PY	FC	PC	LSCS	TA	UC	UD
Prediction accuracy using	Pedigree-based BLUP	0.507	0.389	0.330	0.693	0.684	0.421	0.451	0.477	0.336
	ssGBLUP	0.549	0.450	0.463	0.724	0.745	0.454	0.523	0.473	0.423
	ssGBLUP_*SOCS2*_	0.550	0.450	0.465	0.724	0.745	0.456	0.523	0.473	0.424
	WssGBLUP_(classical, 1)_	0.498	0.422	0.437	0.723	0.730	0.421	0.538	0.473	0.452
	The best WssGBLUP_(m, n)_ method	0.561	0.461	0.486	0.739	0.762	0.471	0.538	0.504	0.460
	Pedigree-based Gene Content	0.557	0.430	0.405	0.698	0.688	0.438	0.448	0.512	0.366
Gain in prediction accuracy between	Pedigree-based BLUP & ssGBLUP	8.25%	15.54%	40.34%	4.41%	8.95%	7.80%	15.84%	-0.96%	26.07%
	ssGBLUP & the best WssGBLUP method	2.16%	2.46%	5.04%	2.06%	2.32%	3.77%	2.80%	6.59%	8.75%
	Without & with the *SOCS2* SNP among the markers (average within the WssGBLUP_(m, n)_ methods)	0.22%	0.13%	0.51%	0.02%	0.10%	1.06%	-0.02%	0.02%	0.26%
	Pedigree-based BLUP & pedigree-based Gene Content	8.88%	9.54%	18.64%	0.68%	0.57%	3.80%	-0.89%	6.72%	8.33%
Parameters of the best WssGBLUP_(m, n)_ method		Maximum 100	Maximum 200	Maximum 200	Maximum 40	Maximum 45	Mean 200	Classical	Sum 30	Maximum 5

*Abbreviations*: *MY* Milk Yield, *FY* Fat Yield, *PY* Protein Yield, *FC* Fat Content, *PC* Protein Content, *LSCS* Somatic Cell Score, *TA* Teat Angle, *UC* Udder Cleft, *UD* Udder Depth

**Fig. 2:**
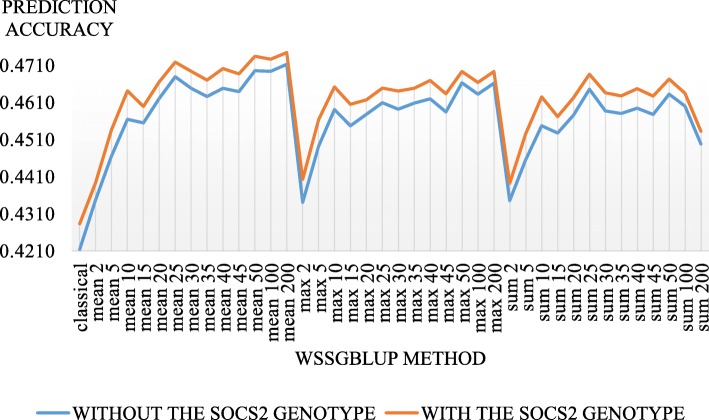
Prediction accuracies of genomic selection from the second iterations of the different WssGBLUP methods for the LSCS trait with (orange curve: WssGBLUP_*SOCS2* (m, n)_) and without (blue curve: WssGBLUP_(m, n)_) the *SOCS2* genotype. Four approaches to the WssGBLUP were computed (m = classical, mean, maximum or sum). The classical WssGBLUP approach (m = classical) gives a different weight for each marker of the chip. In alternative approaches, the chip is decomposed into non-overlapping windows of n markers (we tested *n* = 2, 5, 10, 15, 20, 25, 30, 35, 40, 45, 50, 100, and 200) and within these windows, all markers are assigned the same weight: the mean weight of the n SNPs (m = mean), the maximum weight of the n SNPs (m = maximum), and the sum of the n SNPs weights (m = sum)

In our study, the highest accuracies were obtained by using alternative WssGBLUP approaches. Figure 2 indicates the prediction accuracies for the LSCS trait (as example) with the various WssGBLUP methods. The average gain in accuracy between WssGBLUP methods with and without the *SOCS2* genotype was + 1.06% and the best accuracy (0.471) was obtained with the WssGBLUP_(Mean, 200)_ method.

The gain in prediction accuracy from pedigree-based BLUP to ssGBLUP, the currently used genomic method, was on average + 14.03% (Table 2). An average gain in prediction accuracy of + 3.99% was obtained from ssGBLUP to the best WssGBLUP method (for each trait independently). The average gain in prediction accuracy between all the WssGBLUP methods and WssGBLUP_*SOCS2*_ was + 0.26%. The highest gain (+ 1.06%) was obtained for LSCS.

### Genetic trends of EBVs relative to *SOCS2* and polygenic components using the Gene Content method

The average gain in prediction accuracy between pedigree-based BLUP and pedigree-based Gene Content was + 6.25%. This gain represents the improvement in prediction accuracy for a trait when genome-wide data (54 K herein) are not available and information about a point mutation is known and is included in a multi-trait genetic evaluation model.

**Fig. 3:**
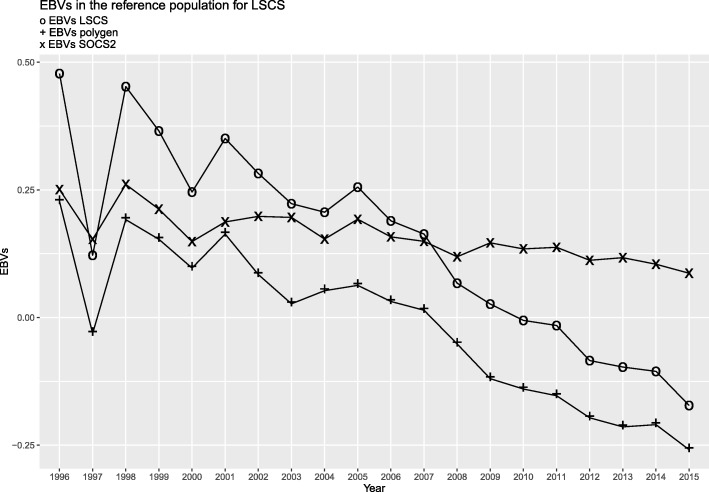
Genetic trends over the years in the reference population (5343 rams with reference performances, i.e., daughter yield deviations, born between 1996 and 2015), of the EBVs for LSCS using the Gene Content method which enables the polygenic component (EBV_*polygen*_), excluding the effect of the *SOCS2* gene, and the breeding value due to the gene effect (EBV_*SOCS2*_), to be distinguished

The Gene Content method was used to obtain EBVs for the polygenic component (EBVs_*polygen*_), excluding the effect of the *SOCS2* gene, EBVs associated with the *SOCS2* gene (EBVs_*SOCS2*_), as well as estimated breeding values for the trait of interest (EBVs_*trait*_), for each trait.The EBVs_*polygen*_ and EBVs_*trait*_ values were very similar for all the traits (Spearman correlation of 0.99), except for LSCS (Spearman correlation of 0.87), the trait most associated with *SOCS2*. The genetic trends of both the polygenic (EBV_*polygen*_) and gene (EBV_*SOCS2*_) components of the LSCS trait over the years are provided in Fig. 3. This graph shows a strong decrease in the EBVs for LSCS since 2004. This decrease has been due partly to the reduced effect of the *SOCS2* gene mutation on the trait (decreased frequency of the deleterious allele), but also to the reduction (improvement) of the polygenic component determining the trait.

### SNP effects estimated by using WssGBLUP methods

The estimated SNPs effects and percentages of the explained variance were determined by applying WssGBLUP methods, with and without the *SOCS2* genotype among the markers. The QTL regions (positions on ovine genome assembly v4.0) found by applying the best alternative WssGBLUP method for each trait (*SOCS2* SNP included in the markers), based on a threshold of 1% of genetic variance explained, are presented in Table 3. According to the SNPs effects (Additional file 1: Figure S4) and the explained variances (Additional file 1: Figure S5), the QTL in the *SOCS2* gene region (Table 3) was confirmed for LSCS, with 20 adjacent SNPs (including the *SOCS2* SNP) explaining as much as 12.00% of the genetic variance. Moreover, this region also influenced PY (4.91% of the variance explained), UD (4.02%), MY (3.94%), FC (2.57%), and UC (1.84%). In addition, among the surrounding SNPs, the *SOCS2* SNP exhibited the strongest (or second strongest) effect (Additional file 1: Figure S4).

**Table 3:** QTL (Quantitative Trait *Loci*) regions (positions on ovine genome assembly v4.0) found using the best alternative WssGBLUP method for each trait (*SOCS2* SNP included among the markers), and based on a threshold of 1% of genetic variance explained

OAR	QTL region (Mb)	Trait associated with the QTL	Genetic variance explained in the trait-specific QTL region^a^ (%)	Trait-specific QTL region^a^ (Mb)
3	128.3 - 130.5	LSCS	12.00	129.1 - 130.5
		PY	4.91	129.0–130.3
		UD	4.02	129.1–130.5
		MY	3.94	128.3–129.6
		FC	2.57	129.1–130.5
		UC	1.84	129.1–130.5
	136.3 - 137.6	PC	4.61	136.3–137.3
		FC	1.20	136.4–137.6
	140.1 - 141.5	PC	1.24	140.1–141.5
6	84.7 - 85.8	PC	5.95	84.7–85.8
11	33.1 - 34.9	FC	6.65	33.4–34.9
		PY	2.25	33.3–34.5
		LSCS	1.98	33.3–34.5
		MY	1.31	33.1–34.3
13	63.4 - 64.5	FC	1.17	63.4–64.5
17	8.5 - 10.5	MY	1.14	8.5–9.7
		FC	1.09	8.6–9.9
		PC	1.02	9.0–10.5
19	44.5 - 45.6	UC	2.33	44.5–45.6
20	48.8 - 49.8	LSCS	3.09	48.8–49.8
23	32.4 - 33.9	UC	2.01	32.4–33.9

*Abbreviations*: *OAR Ovis ARies*, *QTL* Quantitative Trait *Loci*, *Mb* Megabase, *MY* Milk Yield, *FY* Fat Yield, *PY* Protein Yield, *FC* Fat Content, *PC* Protein Content, *LSCS* Somatic Cell Score, *TA* Teat Angle, *UC* Udder Cleft, *UD* Udder Depth

^a^Trait-specific QTL regions are regions where 20 adjacents SNPs explain the highest value of genetic variance of the trait

Several other QTLs were also detected in this study (Table 3). Some of them were trait-specific, such as QTLs on OAR 3 (140.1–141.5 Mb) and 6 (84.7–85.8 Mb), associated with PC, on OAR 19 (44.5–45.6 Mb) and 23 (32.4–33.9 Mb), associated with UC, and on OAR 13 (63.4–64.5 Mb) and 20 (48.8–49.8 Mb), associated with FC and LSCS, respectively. The other three QTLs seemed to be associated with several traits, such as QTLs on OAR 3 (136.3–137.6 Mb), associated with PC and FC, on OAR 11 (33.1–34.9 Mb), associated with MY, PY, LSCS and FC, and on OAR 17 (8.5–10.5 Mb), associated with MY, FC and PC.

## Discussion

### Genetic parameters estimation

The genetic parameters estimation with pedigree or genomic relationships gave similar results for the one-trait methods. In this study the obtained heritabilities were higher for all the traits than previously found in the Lacaune breed. The heritability for LSCS (*h*^2^ = 0.17 − 0.18 in our study) was previously estimated at between 0.12 and 0.15 by Barillet et al. (2001), Rupp et al. (2003) and Barillet et al. (2007) [20–22]. These authors also reported lower heritabilities for MY, FC and PC (0.28–0.34, 0.41–0.50 and 0.51–0.63, respectively). Similar lower results were found for FY and PY (0.26 and 0.28, respectively) [22], and also for the three udder-type traits (0.33–0.35, 0.26–0.32 and 0.19–0.26, for TA, UC and UD, respectively) [22, 23]. These discrepancies could be due, at least in part, to the model as previous studies were based on sire models whereas we used animal models. Other explanations include increased genetic variance within the population (good management of matings in farms, for example) and/or decreased environmental variance (possibly due to the homogenization of breeding practices, for example).

### Detection of QTLs and quantification of their effect using genomic evaluation methods

Our study shows that genomic evaluation methods, which involve weighted approaches and thus an initial step to estimate the effects of SNPs on different traits, can be applied to detect and confirm QTLs [13, 14]. This approach is preferable to a single SNP GWAS (Genome-Wide Association Study) approach because the phenotypes of ungenotyped animals can be directly incorporated, without computing pseudodata, as suggested by Wang et al. (2012) [13].

We initially validated, as expected, the QTL for LSCS on chromosome 3 associated with the *SOCS2* gene point mutation first discovered in Lacaune sheep by Rupp et al. (2015) [19]. When we applied WssGBLUP approaches, this region was found to explain 12% of the genetic variance, as already reported by Rupp et al. (2015) [19]. We also confirmed the pleiotropic effect of this region and its association with milk production traits and UD and were able to quantify its effects on these traits, by applying WssGBLUP approaches. Indeed, this region was found to explain 6.2% of the genetic variance for MY, compared to the 4.4% estimated by Rupp et al. (2015) [19]. The association of this *locus* with UD found in our study might be explained by an indirect effect of individual body size.

We then discovered another pleiotropic QTL on chromosome 11 (33.1–34.9 Mb). This QTL was associated with milk production traits and LSCS, with 1.31, 2.25, 6.65 and 1.98% of the genetic variance explained for MY, PY, FC and LSCS, respectively. This region had previously been associated with LSCS in Lacaune sheep [19] (35.8–41.3 Mb, ovine genome assembly v3.1). A similar pleiotropic QTL was found very near to the orthologous region on caprine chromosome 19 in Saanen goats. Indeed, Martin et al. (2018) [24] reported a pleiotropic QTL (chromosome 19: 24.5–26.9 Mb, caprine genome assembly CHIR_1.0) for milk production and udder traits including MY, FY, PY, udder floor position, and rear udder attachment. This QTL was validated by Teissier et al. (2019) [17] (top 10 SNPs with the highest SNP weights on chromosome 19 located between 26 and 28 Mb, caprine genome assembly CHIR_1.0). It was then confirmed by Oget et al. (2018) [25] (chromosome 19: 22.8–28.9 Mb, caprine genome assembly ARS1) who found that this QTL was also associated with the lifespan of livestock and semen production. However, due to the large number of genes present in the region (47 protein-coding genes, NCBI ovine genome assembly v4.0), proposing suitable candidate genes remains difficult.

In addition to the QTLs associated with LSCS on chromosomes 3 and 11, we also found a QTL on chromosome 20 (48.8–49.8 Mb) that explained 3.1% of the genetic variance. This QTL had previously been detected in sheep [19] (48.6–48.8 Mb, ovine genome assembly v3.1). Sixteen protein-coding candidate genes in this region, two of them related to immune defense (*SERPINB1*, *RIPK1*), were annotated. These three QTLs, associated with LSCS in our study, accounted for as much as 17% of the genetic variance.

Regarding PC, a QTL explaining 5.95% of the genetic variance of this trait was detected on chromosome 6 in a narrow region (84.7–85.8 Mb) known as the casein gene cluster. This region encodes for the caseins (*CSN1S1*, *CSN2*, *CSN1S2*, *CSN3*: 85.0–85.2 Mb), which are the main proteins in milk. Caseins are responsible for milk coagulation, a fundamental step in the preparation of cheese from raw milk. Previous association studies based on microsatellite markers had already highlighted an association of the ovine chromosome 6 with PC, suggesting the role of casein genes, but the confidence intervals obtained with those low-density marker panels were very large [26].

### Weighting SNPs improves genomic evaluation by capturing QTL regions

In 2015, genomic selection was implemented in Lacaune dairy sheep following two comparative studies of evaluation accuracy involving different approaches. Duchemin et al. (2012) [10] compared BLUP, Bayes Cπ, Partial Least Squares (PLS), and sparse PLS methods and reported that including markers in the models increased EBV accuracies by 18 to 25%, depending on the trait (MY, FC et SCS), with minor differences between the genomic approaches. Based on these results, Baloche et al. (2014) [11] adopted BLUP-like methods to implement a single-step model in the evaluation. These authors compared three strategies: pseudo-BLUP (using all rams and DYDs), pseudo-ssGBLUP (using all rams and DYDs), and regular ssGBLUP (using all phenotypes and pedigree in an animal model) and obtained the best results with regular ssGBLUP. Based on these results, the ssGBLUP method is now used for the routine evaluation of Lacaune dairy sheep [11], and hence was the reference method adopted in our study. Using the same ssGBLUP method, we obtained better evaluation accuracies than those of the two previous studies for production traits: + 14.4%, + 1.9% and + 6.1% for MY, FC and PC, respectively. One explanation for this result could be the larger size of the population of genotyped individuals. Indeed, 2892 individuals were genotyped in Baloche et al. (2014) compared to 9844 genotyped animals in our study. However, we obtained lower accuracies for LSCS and the three udder-type traits than Baloche et al. (2014) [11]. No straightforward explanation has been found for this surprising result.

Weighting alternative strategies in the evaluation models was found to provide more accurate results than ssGBLUP for all the traits in our study, with an average gain of + 3.99%, even when no QTL was detected for the trait (teat angle, for example). These results are in slight disagreement with those obtained in goat [17] where the addition of a weighting strategy increased accuracies only for traits that exhibited QTLs. Indeed, the large pleiotropic QTL on chromosome 19 in the Saanen breed [17] allowed an increase in accuracy while in the Alpine breed, with no QTL segregating for most of the traits, WssGBLUP did not provide any significant gain. This disagreement might be explained by the fact that we retained the best alternative method for each trait and did not use the same strategy for all traits, as was done in goats. Indeed, Teissier et al. (2019) [17] used a window size of 40 SNPs for all traits. In our study, depending on the trait, the best accuracies were obtained by using an alternative WssGBLUP strategy with a large window size (100–200 SNPs) for MY, FY and PY, and for LSCS, a medium window size (40–45 SNPs), for FC and PC and a small window size (1–30 SNPs), for the udder-type traits (TA, UC, and UD). Due to the differences in genetic determinism of each trait, (confirmed by the estimated effects of the SNPs in this study, Additional file 1: Figure S4), and possible variation in QTL size, it seemed more appropriate to consider an optimal evaluation strategies for each trait individually. With fine-tuning for each trait, WssGBLUP always proved better than non-weighted ssGBLUP. This highlights a means of increasing genetic progress by taking QTLs into account during genomic evaluation. A further advantage of this method is that no identification of the QTLs, in a dedicated preliminary study, is required.

### Accounting for causal mutations in genomic evaluations

As a case study, we tested different strategies to include the effect of a known causal mutation (*SOCS2* gene) in genomic evaluations. The gain between methods, with and without the mutation in the chip, was limited: an average gain of + 0.26% and up to + 1.06% for LSCS, the trait most influenced by the mutation. This result suggests that the strong LD between the mutation and the surrounding SNPs (0.17) was sufficient to allow accurate estimation of the genomic breeding values without the genotype at the causal mutation, as stated in Goddard (2009) [27]. Including causal SNPs might be of greater interest in the case of lower SNP density and LD around the QTLs or in the case of a stronger effect of the causal mutation.

By applying the Gene Content method, the genetic value (EBV) for a trait can be separated into a genetic value resulting from the effect of a given gene, and a polygenic component resulting from all the other QTLs. In this study, the frequency of the *SOCS2* SNP allele was found to decrease for AI rams (from 0.21 in 2005 to 0.09 in 2017), which might be explained by the introduction of the SCS trait, considered as a proxy for mastitis, into the breeding objectives in Lacaune dairy sheep in 2005 [28, 29]. Indeed, the current relative weight for SCS is 25% in the total merit index. In addition, breeding companies are advised not to retain individuals that are homozygous for the mutation, due to its very negative effect on health. In our study, the slope (Fig. 3) of the decrease over time in the breeding value due to the *SOCS2* gene effect is less pronounced than that due to the polygenic effect. It can therefore be deduced that decreasing the allele frequency of the *SOCS2* mutation over time in the population (unfavourable for the SCS trait) (Fig. 1) does not prevent a favourable trend in other mastitis resistance genes. Thus, in Lacaune sheep, applying a weighting strategy to markers that are strongly associated with the trait of interest (SCS), as with the WssGBLUP approaches, will improve accuracy and therefore response to selection, and may be sufficient to increase mastitis resistance in the whole population.

Two other alternatives to the methods tested in this study would be (i) to include the causal mutation (or major gene) in the evaluation model as a fixed effect [30] and (ii) to use a mixed inheritance model [31]. These alternatives would require knowledge of the gene of interest for a large majority of individuals with a given phenotype, which was not the case in our study. Indeed, the single-step approach makes it possible to start from raw phenotypes and therefore from a very large number of observations (3,575,614 lactations for MY for example). Considering that *SOCS2* information was available for only 9844 individuals (including 1517 females with phenotypes at best), the number of missing data was too large to apply this method to our study.

The presence of a major gene raises two questions: (1) is the polygenic breeding value overestimated when the major gene is ignored [32], (2) what is the risk of reducing total genetic variance over generations of selection [33]. The Gene Content method offers a solution to both questions because it allows the genetic component due to the major gene to be separated from the remaining polygenic component. It might then be possible to manage these two values separately according to the selection objectives i.e., eradicate or fix a major gene allele while maintaining or increasing the polygenic component associated with the trait. The Gene Content method is promising because it allows the genetic variability, i.e. other QTLs or regions with low effects on the same trait, to be preserved in the population, whether the major gene has a stronger effect than the polygenic component for trait prediction or not. This method is also of interest if the gene has a pleiotropic effect on both selected and non-selected traits e.g., a mutation with a favourable effect on a production trait but associated with defects or disease not included in the breeding scheme.

## Conclusions

This study highlights the interest of weighted alternative methods (WssGBLUP) for capturing QTL and major genes in genetic evaluation models. These alternatives increase the accuracy of the predicted genetic values and therefore the expected genetic gains in the population. On the other hand, another approach (Gene Content) tested in this study showed promise for the genetic management of particular traits since it allows the genetic component due to a major gene, to be dissociated from the remaining polygenic component. This latter method is also interesting for populations that have not been genotyped with SNP chips but for which information about a major gene is available. The results of this study pave the way for an improved management of trait genetics, directly applicable to the selection schemes in different livestock sectors.

## Methods

### Animals and phenotypes

The performances of Lacaune sheep registered in the French official milk recording scheme since 1960 and available from the national database (Centre de Traitement de l’Information Génétique, CTIG, Jouy-en-Josas, France) were used for this investigation. The corresponding pedigree information was obtained from the official livestock data system (Ministerial Order NOR: AGRT1431011A, 24th March 2015, Ministry of Agriculture, France).

Nine traits, included in routine genetic evaluations [28, 29, 34], were considered: milk, fat and protein yields, fat and protein contents, somatic cell score (SCS) and three udder-type traits: teat angle, udder cleft, and depth.

The first three lactations were retained for traits related to milk production and SCS. Briefly, milk yield was measured monthly. Milk yield per lactation (MY) was estimated using the Fleischmann method and adjusted for milking length over a reference period of 220 days [35]. SCC, fat and protein contents were measured three times per lactation on average. Lactation traits for fat (FC) and protein (PC) contents were defined as the weighted mean of test days adjusted for milk. Weights were defined according to lactation length and parity. Lactation traits for fat (FY) and protein (PY) yields were the product of MY and corresponding FC and PC. Test-day SCC were log-transformed to somatic cell score (SCS = log_2_(SCC/100) + 3) [36] to normalize the data distribution and were averaged per lactation to compute the analyzed trait LSCS, as described in Rupp et al. (2003) [21].

During the first lactation, three udder-type traits including teat angle (TA), udder cleft (UC) and udder depth (UD) were scored over a linear range of 1 to 9, as described in Marie-Etancelin et al. [23].

Performances were available for ewes since birth years 1978 (MY), 1987 (milk composition traits), 1999 (LSCS) and 2000 (udder-type traits). Data were included up to the birth year 2015. Descriptive statistics for the performance and pedigree files of each trait are given in Table 4.

**Table 4:** Description of the Lacaune dairy sheep dataset used for genetic evaluation

Trait	Mean ± SD	Number of			
		Ewes	Lactations^a^	Individuals in the pedigree file	Rams in the validation population^b^
MY (L)	292.91 ± 85.46	1,503,148	3,575,614	1,651,901	264
FY (g)	213.14 ± 51.56	1,124,636	1,841,351	1,336,060	263
PY (g)	169.72 ± 39.33				
FC (g/L)	66.54 ± 8.51				
PC (g/L)	52.89 ± 4.63				
LSCS	3.12 ± 1.56	769,929	1,321,411	1,031,375	263
TA	7.15 ± 1.06	349,134	349,134	349,134	250
UC	5.04 ± 1.26	349,132	349,132	653,908	249
UD	6.42 ± 0.70	349,132	349,132	653,907	253

*Abbreviations MY* Milk Yield, *FY* Fat Yield, *PY* Protein Yield, *FC* Fat Content, *PC* Protein Content, *LSCS* Somatic Cell Score, *TA* Teat Angle, *UC* Udder Cleft, *UD* Udder Depth

^a^MY, FY, PY, FC, PC and LSCS were measured during the first three lactations (lactation average); TA, UC, and UD were measured once during the first lactation

^b^Rams born in 2015 with Estimated Breeding Values (EBVs) and reference performances, i.e. Daughter Yield Deviation (DYD)

### Genome-wide genotyping data

Genotyping data used in genomic evaluations in this study were available for 9844 individuals that had been genotyped with the medium-density Illumina Ovine 54K SNPs chip [37] for the current genomic selection program [12], or as part of former research projects: “SheepSNPQTL”, “Sustainable Solutions for Small Ruminants”, “Roquefort’in”, and “PhénoFinLait”. This genotyping data was derived from Artificial Insemination (AI) rams (*N* = 8327), born between 1996 and 2015, and progeny-tested by the two Lacaune breeding companies (OVI-TEST -La Glène, Saint-Léons, France- and Confédération Générale de Roquefort -Le Bourguet, Vabres l’Abbaye, France-), and from ewes (*N* = 1517), born between 2004 and 2013, and used for QTL detection programs.

DNA extraction from blood samples and genotyping were performed at the Laboratoire d’Analyses Génétiques pour les Espèces Animales (LABOGENA -Jouy en Josas, France-; www.labogena.fr). SNPs were remapped on version 4.0 of the ovine genetic map (https://www.ncbi.nlm.nih.gov/assembly/GCF_000298735.2/). Quality control was performed as part of the routine pipeline for Lacaune genotyped animals, as described in Baloche et al. [11] with slight modifications. In brief, SNPs with a MAF lower than 1%, Hardy Weinberg disequilibrium (*P* < 10^− 5^) and a call rate lower than 97% were removed. After edits, 37,941 out of 54,241 SNPs remained for the analyses.

### *SOCS2* genotypes and imputation of missing data

Genotypes for the point mutation of interest in the *SOCS2* gene (hereafter called *SOCS2* SNP, rs868996547, *Ovis aries* -OAR- chromosome 3, position 129,557,942 on ovine genome assembly v4.0) were available for 4297 animals. The data were derived from two datasets. First, KASPar™ tests (described in Rupp et al. [19]) were obtained as part of the “Sustainable Solutions for Small Ruminants” and “REIDSOCS” (ANR-16-CE20-0010 funded by the ANR -Paris, France) projects for 1413 AI rams and 248 ewes from one INRA (Institut National de la Recherche Agronomique) experimental farm (La Fage -Roquefort-Sur-Soulzon, France-) born during 2002–2003. These 1661 individuals are included in the 9844 individuals genotyped with the 54K SNPs chip presented in the previous section. Second, young rams (*N* = 2636) which entered breeding centers in 2017 were low-density genotyped with the International Sheep Genomics Consortium (ISGC) panel which includes 1500 SNPs [38]. This chip also contains the *SOCS2* mutation as a SNP and suitable genotypes were subsequently extracted. In addition, these rams were imputed from low to 54K density as part of the routine genomic evaluation [39]. All 4297 animals were then genotyped both for *SOCS2* and for the 54K SNPs panel.

The *SOCS2* genotype was then imputed using the FImpute v2.2 software [40] for the 9844 individuals genotyped with the 54K SNPs chip. For this imputation step, individuals from AI centers born in 2016 and genotyped with the 54K SNPs chip were also added to the data set to fill the missing year that connected young males born in 2017 to the rest of the genotyped population. Thus 10,432 out of the total of 14,729 individuals genotyped with the 54K SNPs chip had missing *SOCS2 locus* information and required imputation. A cross-validation test was performed to assess the accuracy of imputation by designing an appropriate validation population for which the *SOCS2* genotypes were ascribed to missing values. The validation population represented one-third of the total number of *SOCS2* genotypes, i.e. 1432 individuals selected at random. The accuracy of imputation was calculated by counting the errors between the true and imputed genotypes in the validation population. The accuracy of the imputation corresponded to the concordance rate (CR), with CR = 1 − error _ rate, where $\mathrm{error}\_\mathrm{rate}=\frac{\mathrm{number}\ \mathrm{of}\ \mathrm{error}\mathrm{s}}{2\times \mathrm{number}\ \mathrm{of}\ \mathrm{imputed}\ \mathrm{individuals}}$.

### Linkage disequilibrium (LD)

After imputing the *SOCS2* genotypes, we then computed the linkage disequilibrium (LD) in the surrounding chromosomal region. Using the Haploview v4.2 software [41], the square correlation coefficient *r*^2^ measure of LD [42] was calculated between each pair of SNPs for *SOCS2* and the 40 closest markers (Additional file 1: Figure S2).

The LD of the *SOCS2* region was then compared with the mean LD of the chip by computing the *r*^2^ between all SNP pairs in 10-Megabase (Mb) windows within the chromosomes. The *r*^2^ were grouped into categories based on the distance between SNP (every 0.02 Mb) (Additional file 1: Figure S1).

### Genomic prediction methods

EBVs were computed for all animals in the pedigree files (Table 4) using the following evaluation approaches. With the first set of methods, a single-trait approach was used for all nine traits of interest. With the second set of methods, a multi-trait approach called Gene Content, developed by Legarra and Vitezica [15], was used in which the *SOCS2* genotype was considered as a trait in bivariate evaluations with MY, FC, PC, FY, PY, LSCS, TA, UC and UD. All methods were based on a single-step process [5], e.g. use of all data from females together with the pedigree, and genomic information if available. These different approaches are summarized in Table 5.

**Table 5:** Description of the different genetic evaluation models based on a single-step approach and using information about the *SOCS2* gene or not

Approach	Model	Use of *SOCS2* data	Information used in the relationship matrix		
			Pedigree	54K SNPs	*SOCS2* SNP
Single-trait	Pedigree-based BLUP	No	Yes	No	No
	ssGBLUP	No	Yes	Yes	No
	ssGBLUP_*SOCS2*_^a^	Yes	Yes	Yes	Yes
	WssGBLUP_(m, n)_^b^	No	Yes	Yes	No
	WssGBLUP_*SOCS2* (m, n)_^b^	Yes	Yes	Yes	Yes
Multiple-trait	Pedigree-based Gene Content	Yes (as a trait)	Yes	No	No

*Abbreviations*: *GBLUP* Genomic Best Linear Unbiaised Prediction, *ss* single-step, *W* Weighted

^a^The term *SOCS2* here means that the *SOCS2* SNP has been added to the 54K SNPs of the chip

^b^Four approaches to the WssGBLUP were computed (m = classical, mean, maximum or sum). The classical WssGBLUP approach (m = classical) gives a different weight for each marker of the chip. In alternative approaches, the chip is decomposed into non-overlapping windows of n markers (we tested n = 2, 5, 10, 15, 20, 25, 30, 35, 40, 45, 50, 100, and 200) and within these windows, all markers are assigned the same weight: the mean weight of the n SNPs (m = mean), the maximum weight of the n SNPs (m = maximum), and the sum of the n SNPs weights (m = sum)

#### Single-trait approaches

For single-trait approaches, we applied the following model (1) to the five milk production traits (MY, FC, PC, FY, and FC) and LSCS:

1 $$ y= X\beta + Zg+ Wp+\varepsilon $$

where *y* is the observation vector for the trait (female lactation performances) and *β* is a vector of fixed effects. The fixed effects for each trait are listed in Table 6. *g* is a vector of random additive genetic effects assumed to be normally distributed $N\left(0,H{\hat{\sigma}}_g^2\right)$, with *H* the relationship matrix. *p* is a vector of random permanent environmental effects assumed to be normally distributed $N\left(0,I{\hat{\sigma}}_p^2\right)$ and *ε* is a vector of random residuals that is normally distributed $N\left(0,I{\hat{\sigma}}_{\varepsilon}^2\right)$. *X* is the incidence matrix relating phenotypes to the fixed effects (*β*), *Z* is the design matrix allocating phenotypes to breeding values (*g*) and *W* is the incidence matrix relating phenotypes to permanent environmental effects (*p*).

**Table 6:** Description of fixed effects for the evaluation models of each phenotype

Trait	Fixed effects	Number of levels
MY	• herd within year and within parity • age at delivery within year and within parity • month at delivery within year and within parity • time between delivery and first OMR within year and within parity	42259 514 702 585
FY	• herd within year and within parity • age at delivery within year and within parity • quality control within year and within parity	20783 269 348
PY		
FC		
PC		
LSCS	• herd within year and within parity • age at delivery within year and within parity • month at delivery within year and within parity	14306 206 312
TA	• herd within year • interaction between examiner and the time difference between milking and scoring, within herd • interaction between age at delivery and lactation stage, within year • number of lambs within year	3672 2346 160 30
UC		
UD		

*Abbreviations*: *MY* Milk Yield, *FY* Fat Yield, *PY* Protein Yield, *FC* Fat Content, *PC* Protein Content, *LSCS* Somatic Cell Score, *TA* Teat Angle, *UC* Udder Cleft, *UD* Udder Depth

Since the three udder-type traits had only one record per female, we removed the random permanent environmental term from the eq. (1) and applied the following model with the same parameters as in (1):

2 $$ y= X\beta + Zg+\varepsilon $$

In the pedigree files, we added 24 unknown parent groups defined as follows: animals born before 1960, cohorts born within 10-year windows up to 2000, cohorts born within 5-year windows up to 2010, cohorts born within 2-year windows up to 2014, and finally animals born in 2015.

We modeled the relationship matrix *H* using the different approaches summarized in Table 5. Briefly, only pedigree information was used for the pedigree-based BLUP and a combination of pedigree and genomic information for the ssGBLUP method, as described in Legarra et al. [5].

Next, four WssGBLUP methods were applied. These differed from the ssGBLUP method in that the genetic relationship matrix was altered by weighting the chip markers, the weights being iteratively derived from the decomposition of EBVs into marker effects. Indeed, SNP effects can be deduced from EBVs in the genomic-based single-trait approaches (eqs. (1) and (2)), as modeled in Wang et al. (2012) [13]: $\hat{a}=\mathrm{DM}\hbox{'}{\left[\mathrm{MDM}\right]}^{-1}{\hat{g}}_{gen}$. In this equation, $\hat{a}$ is a vector of estimated SNP effects, D is a diagonal matrix of weights (set at 1 in the ssGBLUP method), M is the centered matrix of SNP genotypes, and ${\hat{g}}_{gen}$ is the vector of EBVs from genotyped animals only.

Wang et al. [13] showed that WssGBLUP was sufficient, with only very few iterations, to attain a maximum accuracy of EBV. Similarly to Wang et al. (2012), and validated in Teissier et al. [16, 17], the highest prediction accuracies in our study were obtained after two iterations (results not shown) and therefore only the results after two iterations are provided. The decrease in prediction accuracy after the second iteration in the WssGBLUP approaches could be due to excessive weighting of SNPs associated with a few high effect QTLs, and reduced weighting of numerous low-effect QTLs. Alternative weighting methods, that assign the same weight to several adjacent chip markers, have been proposed by Zhang et al. [14]. In our study, we computed three alternative methods using WssGBLUP: (1) the mean weight of n SNPs (with n the number of adjacent SNPs with non-overlapping windows), (2) the maximum weight of n SNPs, and (3) the sum of n SNPs weights. Weights were calculated as described in Teissier et al. [16]. Briefly, calculations of the weights used in the diagonal matrix D in these alternative methods were based on the variances of SNPs effects estimated with the first step ssGBLUP. After assigning the same value (mean, sum or maximum) to the n markers of a window, the vector of marker weights was then normalized so that the sum of all weights remained constant and equal to the total number of SNPs. Several window sizes with varying number of SNPs were used (n = 2, 5, 10, 15, 20, 25, 30, 35, 40, 45, 50, 100, and 200). Hereafter, these methods are designated WssGBLUP_(m, n)_, where m is the method used to calculate the weights and n the number of adjacent SNPs with non-overlapping windows.

Estimates of SNP effects can also be used to estimate the genetic variance of the trait explained by each SNP *i* effect: ${\hat{\sigma}}_{a,i}^2={\hat{a}}_i^22{\hat{p}}_i\left(1-{\hat{p}}_i\right)$, where *p*_*i*_ is the allele frequency of SNP *i*. The explained variances will be described for 20 adjacent SNPs in the result and discussion sections.

#### Multiple-trait gene content approaches

In the Gene Content method [15] EBVs are estimated for a given trait by simultaneously considering information about a given genotype as a second trait (hereafter called the *SOCS2* gene content trait) in a two-trait approach. Accordingly, the following model (3) was applied to the five milk production traits and LSCS:

3 $$ \left\{\begin{array}{l}y= X\beta + Zg+ Wp+\varepsilon \\ {}{y}_T={\mu}_T+{Z}_T{g}_T+{\varepsilon}_T\end{array}\right. $$

where *y* is the observation vector for the trait (female lactation performance) and parameters of the model *β*, *g*, *p*, *ε*, *X* and *W* are the same as in eq. (1). *y*_*T*_ is a vector of the *SOCS2 g*ene content trait, i.e. the number of copies of the mutant T allele carried by each animal (0, 1 or 2). Missing values were set for ungenotyped individuals. *μ*_*T*_ is the mean fixed effect of the *SOCS2* T allele, *Z*_*T*_ is the incidence matrix relating observations to the random genetic effect (*g*_*T*_) of the *SOCS2 g*ene content trait, which was assumed to be normally distributed such that ${\sigma}_{g_T}^2=H{\hat{\sigma}}_{g_T}^2$ and ${\hat{\sigma}}_{gT}^2=2{\hat{f}}_T\left(1-{\hat{f}}_T\right)$, with *f*_*T*_ the T allele frequency, and *ε*_*T*_ the random residual error.

As before, the model (4) was simplified for the three non-repeated udder-type traits:

4 $$ \left\{\begin{array}{l}y= X\beta + Zg+\varepsilon \\ {}{y}_T={\mu}_T+{Z}_T{g}_T+{\varepsilon}_T\end{array}\right. $$

For Eqs. (3) and (4) only the pedigree-based approach was tested, in order to avoid redundant information between the chip and the *SOCS2* gene content trait (copy number of the mutated allele), which meant that the kinship matrix *H* was modeled using only pedigree information (Table 5).

The evaluations were done using BLUP90IOD2 v3.102 software [43].

By applying the Gene Content method, we were able to obtain not only EBVs for the trait of interest ($\hat{g}$), but also estimates of a breeding value for the polygenic component (EBV_*polygen*_) that excluded the effect of the *SOCS2* gene, as well as estimates of breeding values associated with the *SOCS2* gene (EBV_*SOCS2*_). We calculated these three EBVs as proposed by Legarra and Vitezica (2015) [15]: ${\mathrm{EBV}}_{polygen}={\mathrm{EBV}}_{trait\_ of\_ interest}-{\mathrm{EBV}}_{SOCS2}=\hat{g}-{\hat{g}}_T\hat{\alpha}$, with $\hat{\alpha}=\frac{c\hat{o}v\left(g,{g}_T\right)}{{\hat{\sigma}}_{g_T}^2}$, which can be interpreted as the allele substitution effect of the *SOCS2* gene mutation on the trait.

### Variance component estimation

Variance and (co)variance (for the Gene Content method) components for models (1), (2), (3) and (4) for each approach and each trait were estimated using a block implementation of Gibbs sampling computed in the GIBBS1F90 v1.44 software [43].

Based on these variance component estimations, the genetic variance explained by the *SOCS2* gene for each trait was calculated by applying the following equation [15]: $\mathrm{explained}\_\mathrm{variance}=\frac{{\hat{\alpha}}^2\times {\hat{\sigma}}_{g_T}^2}{{\hat{\sigma}}_g^2}$, with *α* the allele substitution effect of the *SOCS2* gene mutation on the trait described previously.

We also used the estimated variance components to derive the genetic correlation (*r*_*g*_) between the *SOCS2* gene content trait and the trait of interest with the following equation:

$$ {\hat{r}}_g=\frac{c\hat{o}v\left(g,{g}_T\right)}{{\hat{\sigma}}_g\times {\hat{\sigma}}_{g_T}}. $$

### Prediction accuracy

To validate the EBVs, we added progeny performances from 264 males born in 2015, which had not been used to predict these EBVs, to a validation set (Table 4). We computed the accuracies of the genomic predictions for each model and for each trait using the Pearson correlation between EBVs for the males in the validation population and DYDs. The numbers of rams in the validation population for each trait with EBVs and DYD are shown in Table 4.

## Additional file

Additional file 1: **Figure S1** Visualization of linkage disequilibrium (*r*^2^ × 100) between the 40 markers closest to the *SOCS2* point mutation (rs868996547, in the middle). **Figure S2** Visualization of linkage disequilibrium measured as squared correlation coefficient (*r*^2^) according to distance between markers on the 50 K ovine SNP chip. **Figure S3** Components estimations according to the different models. One-trait methods correspond to eqs. (1) and (2) and two-traits methods to eqs. (3) and (4). **Figure S4** Manhattan plots of estimated SNP effects using the best WssGBLUP approach for each phenotype (second iteration). On the left are presented analysis without the *SOCS2* genotype among the markers and on the right, with the *SOCS2* genotype (green point). **Figure S5** Manhattan plots of estimated variance explained by 20 adjacent SNPs using the best WssGBLUP approach for each phenotype (second iteration). The horizontal red line represents the threshold of 1% adopted in this study. On the left are presented the analyses without the *SOCS2* genotype among the markers and on the right, with the *SOCS2* genotype. (DOCX 2190 kb)

## Data Availability

The datasets used and analyzed during the current study are available from the corresponding author on reasonable request.

## Abbreviations

AI: Artificial Insemination
ANR: Agence Nationale de la Recherche
BLUP: Best Linear Unbiased Prediction
CR: Concordance Rate
DYD: Daughter Yield Deviation
FC: Fat Content
FEDER: Fonds Européen de Développement Régional
FGE: France Génétique Elevage
FUI: Fonds Unique Interministériel
FY: Fat Yield
GBLUP: Genomic Best Linear Unbiased Prediction
INRA: Institut National de la Recherche Agronomique
ISGC: International Sheep Genomics Consortium
LABOGENA: Laboratoire d’Analyses Génétiques pour les Espèces Animales
LD: Linkage Disequilibrium
MAF: Minor Allele Frequency
MY: Milk Yield
OMR: Official Milk Recording
PC: Protein Content
PY: Protein Yield
QTL: Quantitative Trait Loci
SCS: Somatic Cell Score
SNP: Single Nucleotide Polymorphism
SOCS2: Suppressor Of Cytokine Signaling 2
ssGBLUP: Single-step Genomic Best Linear Unbiased Prediction
TA: Teat Angle
UC: Udder Cleft
UD: Udder Depth
WssGBLUP: Weighted single-step Genomic Best Linear Unbiased Prediction
