# Supplementary material for: Alternative methods improve the accuracy of genomic prediction using information from a causal point mutation in a dairy sheep model
Source: BMC Genomics. 2019 Sep 18;20:719. doi: 10.1186/s12864-019-6068-4 (PMC6751880; doi:10.1186/s12864-019-6068-4)

Additional figures

Figure S1 Visualization of linkage disequilibrium measured as squared correlation coefficient ($r^{2}$) according to distance between markers on the 50K ovine SNP chip.


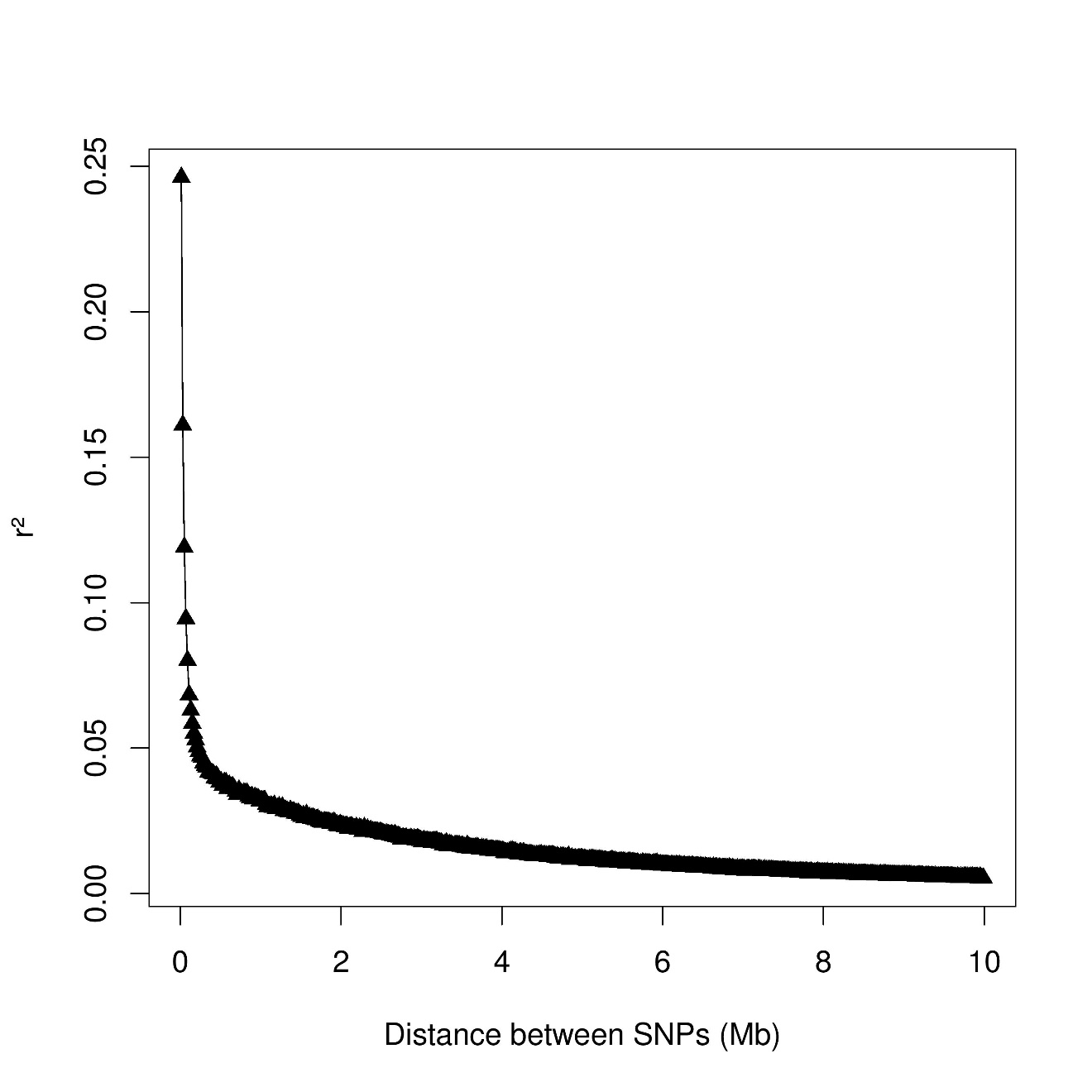


**Figure S2** Visualization of linkage disequilibrium ($r^{2}\times100$) between the 40 markers closest to the *SOCS2* point mutation (rs868996547, in the middle).
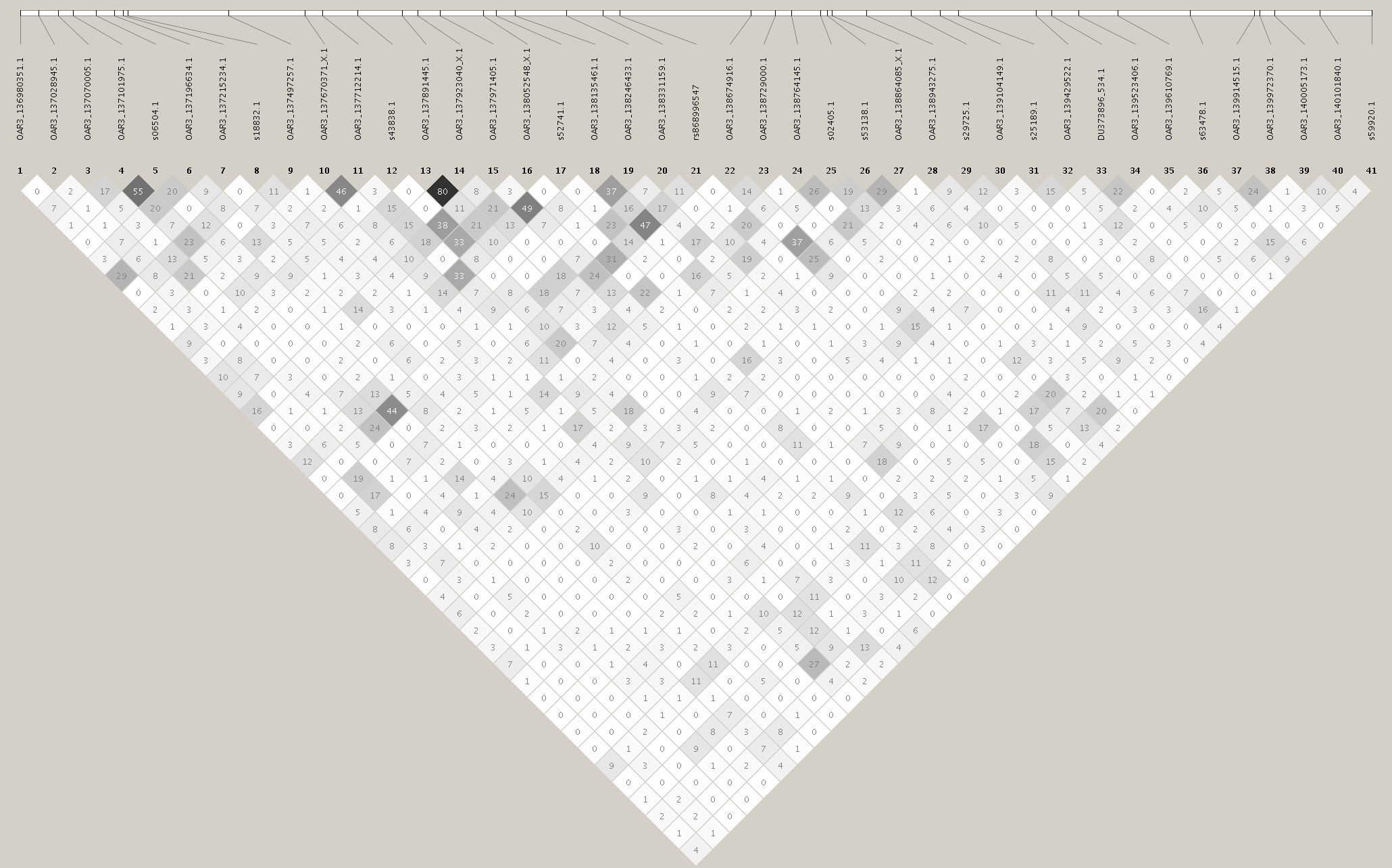


Figure S3 Components estimations according to the different models. One-trait methods correspond to equations (1) and (2) and two-traits methods to equations (3) and (4).

| *Method* | *Component* | *MY* | | *FY* | | *PY* | | *FC* | | *PC* | | *SCS* | | *TA* | | *UC* | | *UD* | |
| --- | --- | --- | --- | --- | --- | --- | --- | --- | --- | --- | --- | --- | --- | --- | --- | --- | --- | --- | --- |
| One-trait  pedigree-  based | $\hat{\sigma}_{g}^{2}$ | 1425.00 ± 1.07 | | 604.17 ± 0.45 | | 327.80 ± 0.24 | | 25.86 ± 0.14 | | 8.63 ± 3.98E-02 | | 0.38 ± 4.78E-03 | | 0.37 ± 5.25E-03 | | 0.45 ± 7.08E-03 | | 0.10 ± 1.61E-03 | |
|  | $\hat{\sigma}_{p}^{2}$ | 977.98 ± 0.65 | | 311.72 ± 0.29 | | 168.43 ± 0.15 | | 4.96 ± 9.41E-02 | | 1.30 ± 2.37E-02 | | 0.33 ± 3.87E-03 | |  | |  | |  | |
|  | $\hat{\sigma}_{\varepsilon}^{2}$ | 1432.00 ± 0.15 | | 716.24 ± 0.12 | | 342.93 ± 5.63E-02 | | 21.26 ± 3.64E-02 | | 4.13 ± 6.93E-03 | | 1.46 ± 2.69E-03 | | 0.58 ± 3.43E-03 | | 0.87 ± 5.15E-03 | | 0.25 ± 1.28E-03 | |
|  | $h^{2}$ | 0.37 | | 0.37 | | 0.39 | | 0.50 | | 0.61 | | 0.17 | | 0.39 | | 0.34 | | 0.27 | |
| One-trait  genomic-  based | $\hat{\sigma}_{g}^{2}$ | 1431.60 ± 1.07 | | 607.51 ± 0.45 | | 329.84 ± 0.24 | | 25.92 ± 0.14 | | 8.66 ± 3.94E-02 | | 0.38 ± 4.73E-03 | | 0.37 ± 5.38E-03 | | 0.45 ± 7.30E-03 | | 0.10 ± 1.67E-03 | |
|  | $\hat{\sigma}_{p}^{2}$ | 975.35 ± 0.65 | | 310.55 ± 0.29 | | 167.68 ± 0.15 | | 4.94 ± 9.00E-02 | | 1.29 ± 2.28E-02 | | 0.33 ± 3.75E-03 | |  | |  | |  | |
|  | $\hat{\sigma}_{\varepsilon}^{2}$ | 1432.00 ± 0.15 | | 716.24 ± 0.12 | | 342.93 ± 5.66E-02 | | 21.26 ± 3.65E-02 | | 4.13 ± 6.93E-03 | | 1.46 ± 2.71E-03 | | 0.58 ± 3.47E-03 | | 0.87 ± 5.23E-03 | | 0.25 ± 1.30E-03 | |
|  | $h^{2}$ | 0.37 | | 0.37 | | 0.39 | | 0.50 | | 0.61 | | 0.18 | | 0.39 | | 0.34 | | 0.28 | |
| Two-traits  pedigree-  based | Covariances  matrix $g$ | 1423.60  ± 0.87 | 45.58  ± 0.10 | 605.45  ± 0.42 | 21.79  ± 0.10 | 328.08  ± 0.22 | 26.25  ± 5.84E-02 | 25.93  ± 0.13 | -0.34  ± 2.16E-02 | 8.66  ± 3.65E-02 | -9.21E-02  ± 1.65E-02 | 0.38  ± 5.03E-03 | 0.10  ± 1.96E-03 | 0.37  ± 5.00E-03 | -5.90E-03  ± 4.68E-03 | 0.45  ± 6.58E-03 | -2.28E-02  ± 7.47E-03 | 0.10  ± 1.87E-03 | -2.70E-02  ± 3.27E-03 |
|  |  | 45.58  ± 0.10 | 23.61  ± 0.11 | 21.79  ± 0.10 | 24.36  ± 0.12 | 26.25  ± 5.84E-02 | 24.56  ± 0.12 | -0.34  ± 2.16E-02 | 0.24  ± 1.19E-02 | -9.21E-02  ± 1.65E-02 | 0.24  ± 9.51E-03 | 0.10  ± 1.96E-03 | 0.25  ± 2.91E-03 | -5.90E-03  ± 4.68E-03 | 0.21  ± 7.78E-03 | -2.28E-02  ± 7.47E-03 | 2.08E-01  ± 5.38E-03 | -2.70E-02  ± 3.27E-03 | 0.20  ± 9.70E-03 |
|  | Covariances  matrix $p$ | 979.00  ± 0.54 | 0 | 310.71  ± 0.26 | 0 | 168.14  ± 0.14 | 0 | 4.91  ± 7.65E-02 | 0 | 1.28  ± 2.04E-02 | 0 | 0.32  ± 4.25E-03 | 0 |  |  |  |  |  |  |
|  |  | 0 | 0 | 0 | 0 | 0 | 0 | 0 | 0 | 0 | 0 | 0 | 0 |  |  |  |  |  |  |
|  | Covariances  matrix $\varepsilon$ | 1432.00  ± 0.15 | 0.37  ± 9.63E-02 | 716.34  ± 0.13 | -0.17  ± 3.99E-02 | 342.98  ± 6.08E-02 | -0.30  ± 2.95E-02 | 21.27  ± 3.58E-02 | 5.62E-03  ± 6.19E-03 | 4.13  ± 7.10E-03 | -6.10E-04  ± 3.05E-03 | 1.45  ± 3.08E-03 | 1.90E-03  ± 3.13E-03 | 0.56  ± 3.42E-03 | -2.88E-03  ± 9.35E-03 | 0.85  ± 4.71E-03 | -1.06E-02  ± 7.11E-03 | 0.24  ± 1.38E-03 | -8.25E-04  ± 6.00E-03 |
|  |  | 0.37  ± 9.63E-02 | 0.66  ± 7.21E-02 | -0.17  ± 3.99E-02 | 0.76  ± 7.90E-02 | -0.30  ± 2.95E-02 | 0.72  ± 7.37E-02 | 5.62E-03  ± 6.19E-03 | 7.43E-03  ± 7.75E-03 | -6.10E-04  ± 3.05E-03 | 5.39E-03  ± 5.52E-03 | 1.90E-03  ± 3.13E-03 | 1.53E-03  ± 2.46E-03 | -2.88E-03  ± 9.35E-03 | 3.67E-02  ± 4.58E-03 | -1.06E-02  ± 7.11E-03 | 3.55E-02  ± 2.97E-03 | -8.25E-04  ± 6.00E-03 | 3.88E-02  ± 5.71E-03 |
|  | $h^{2}$ | 0.37 | | 0.37 | | 0.39 | | 0.50 | | 0.62 | | 0.18 | | 0.40 | | 0.35 | | 0.28 | |

Abbreviations: GC: Gene Content; Gen. SD: Genetic standard deviation between homozygous individuals for the *SOCS2* genotype; MY: Milk Yield; FY: Fat Yield; PY: Protein Yield; FC: Fat Content; PC: Protein Content; SCS: Somatic Cell Score; TA: Teat Angle; UC: Udder Cleft; UD: Udder Depth

Figure S4 Manhattan plots of estimated SNP effects using the best WssGBLUP approach for each phenotype (second iteration). On the left are presented analysis without the *SOCS2* genotype among the markers and on the right, with the *SOCS2* genotype (green point).


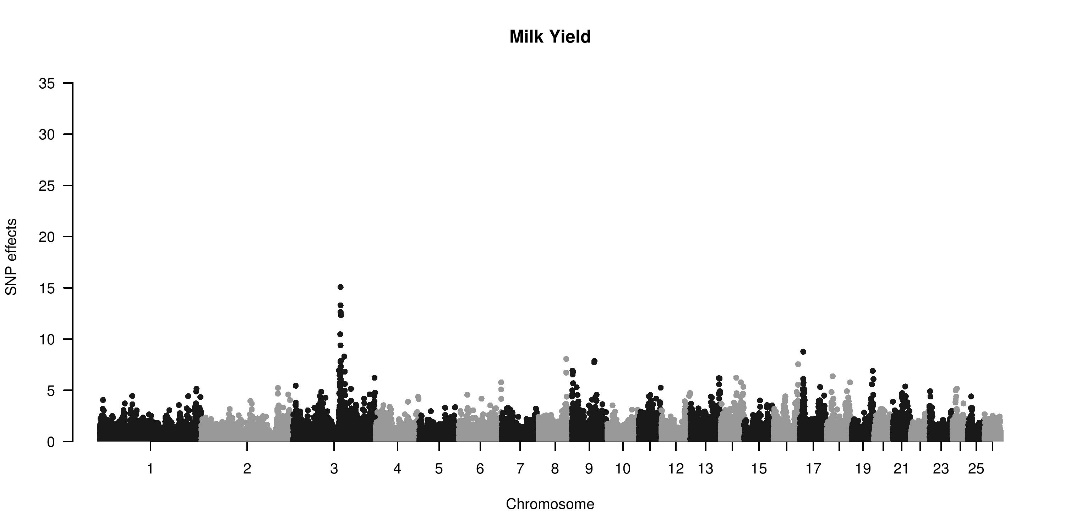

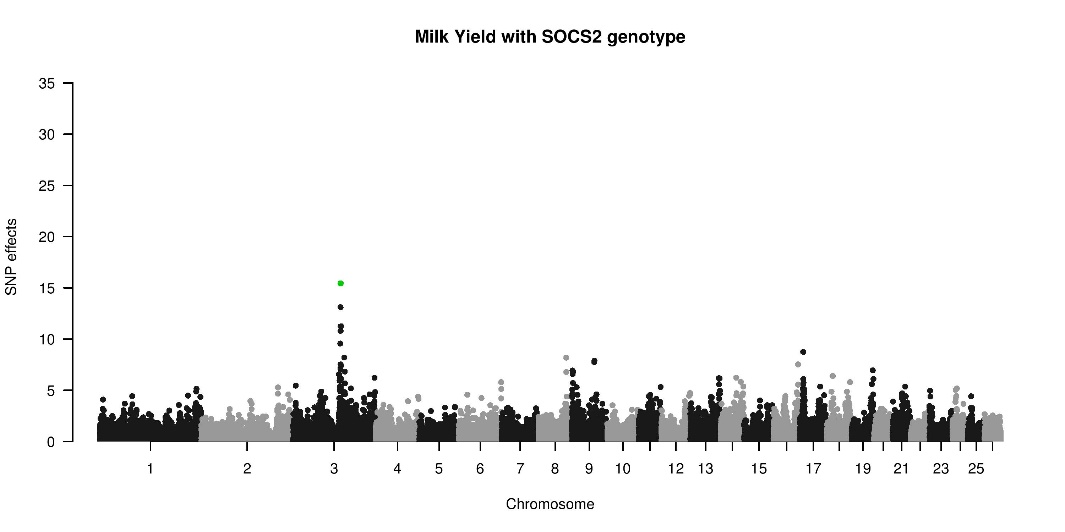

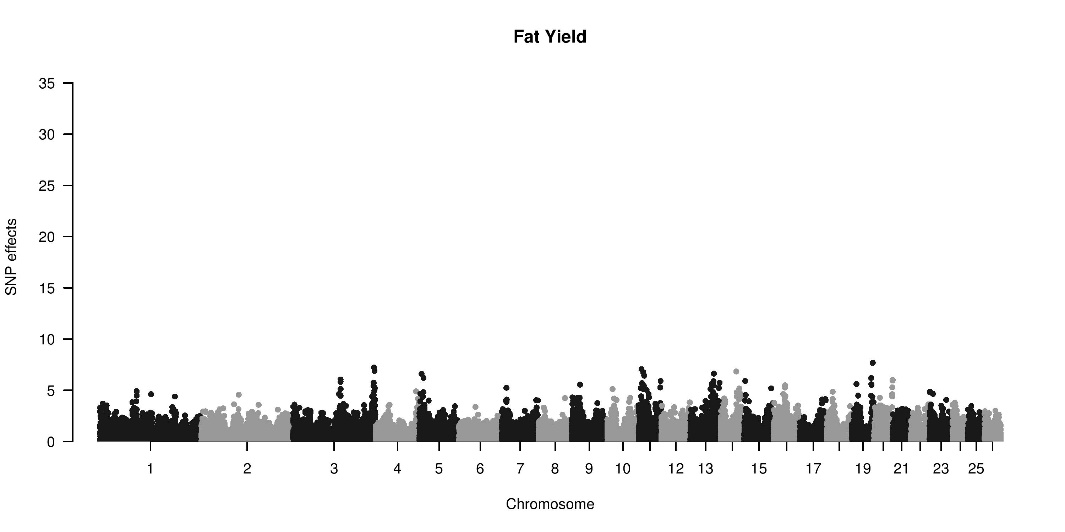

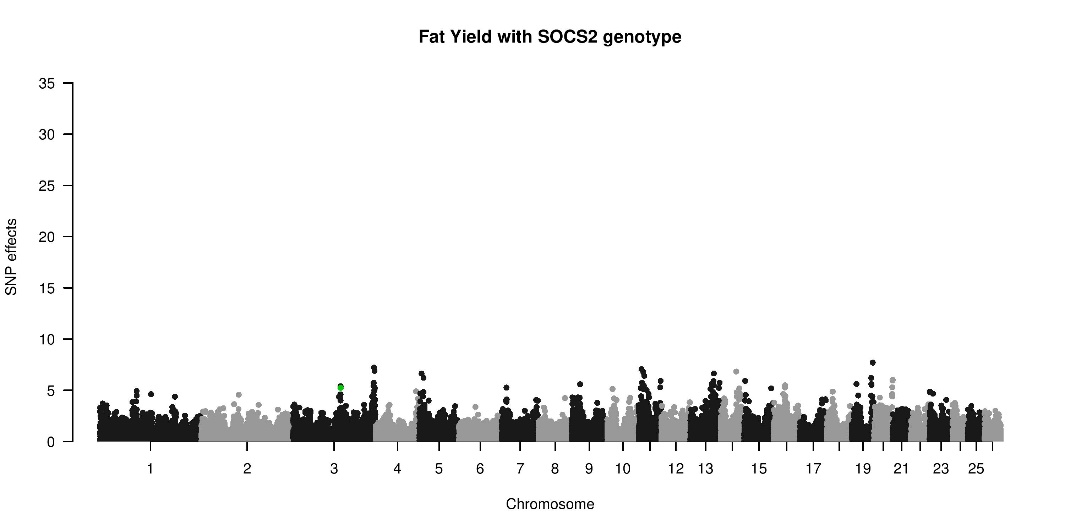

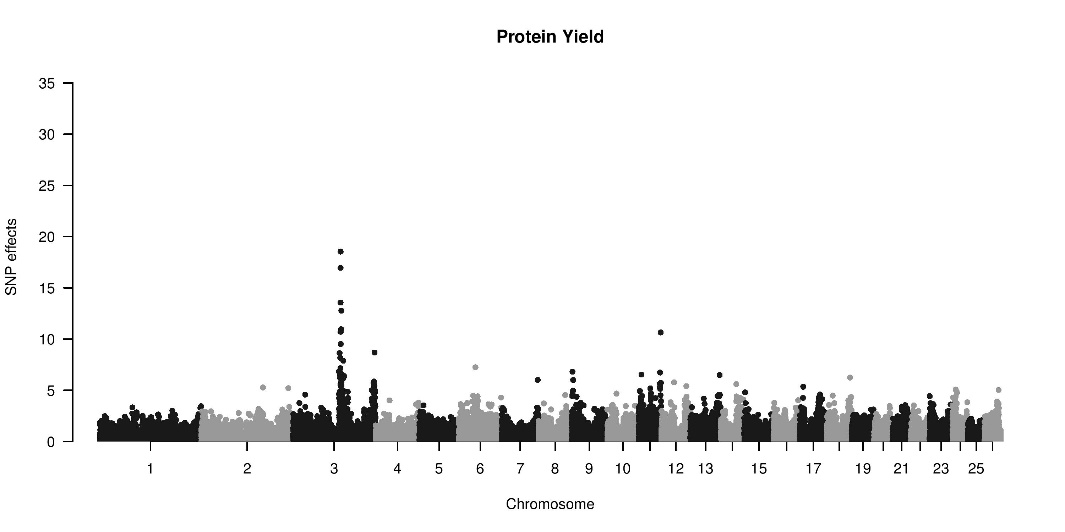

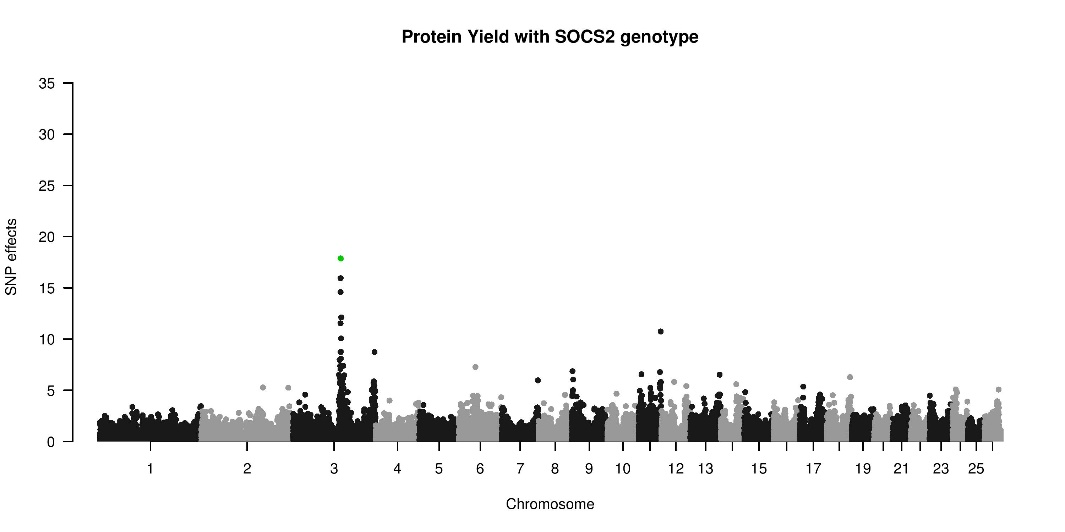

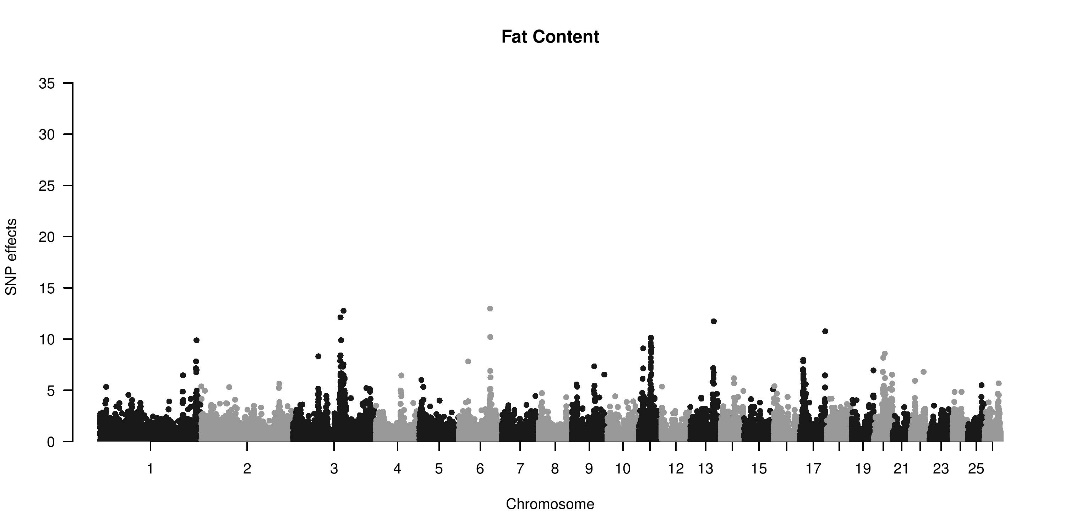

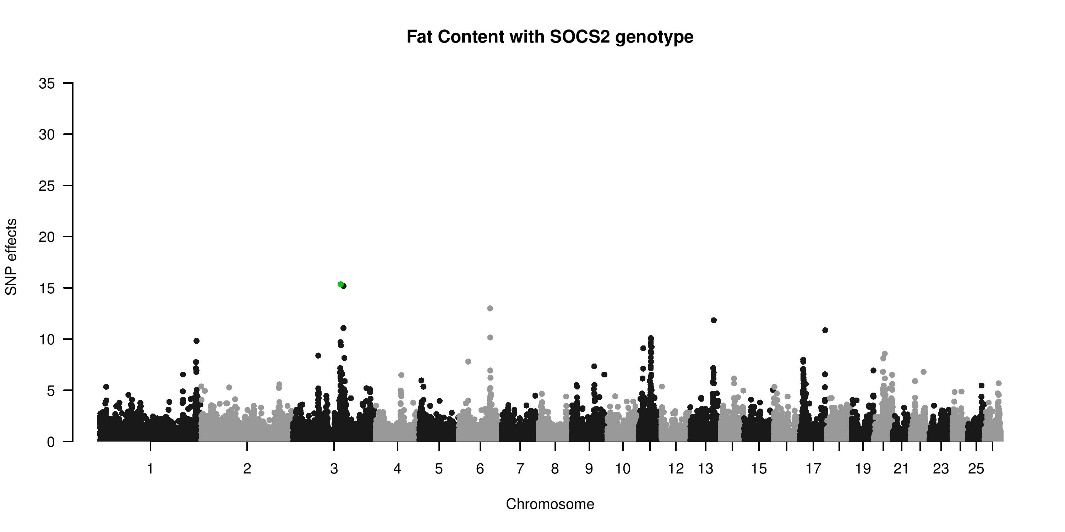

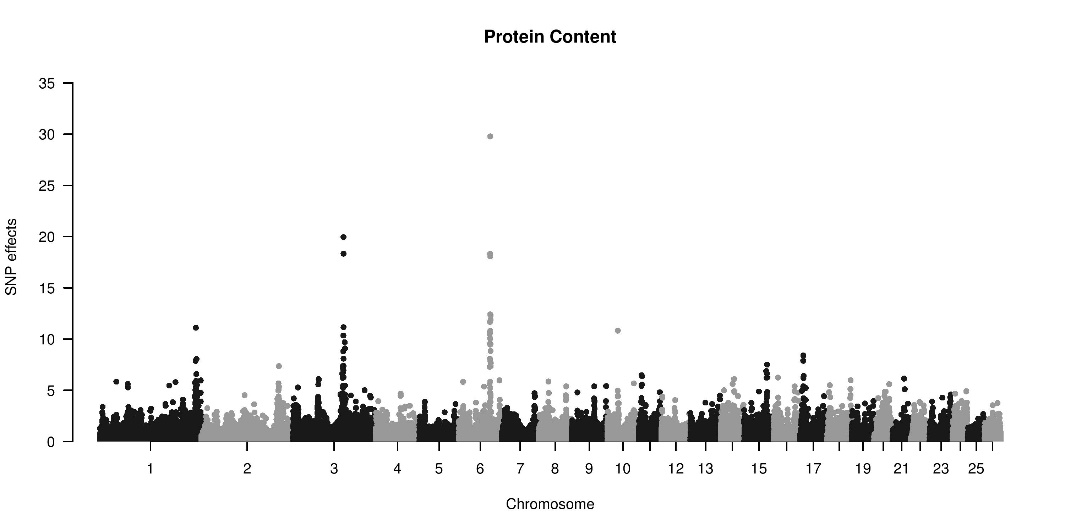

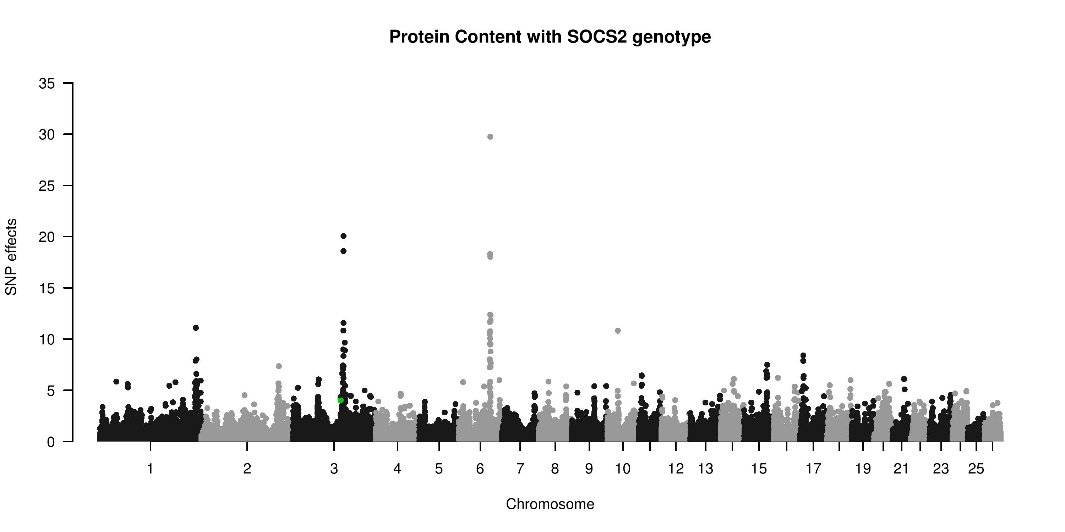


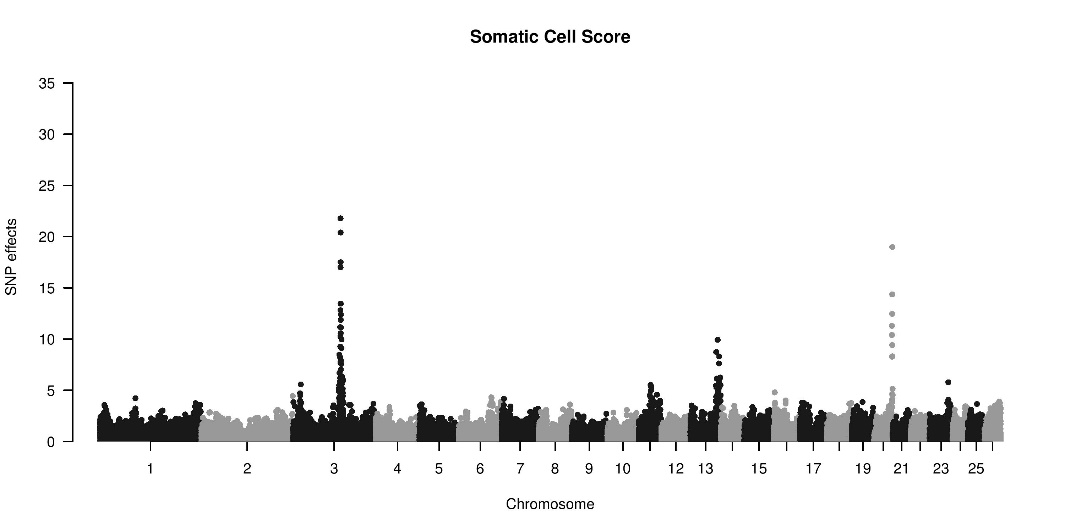

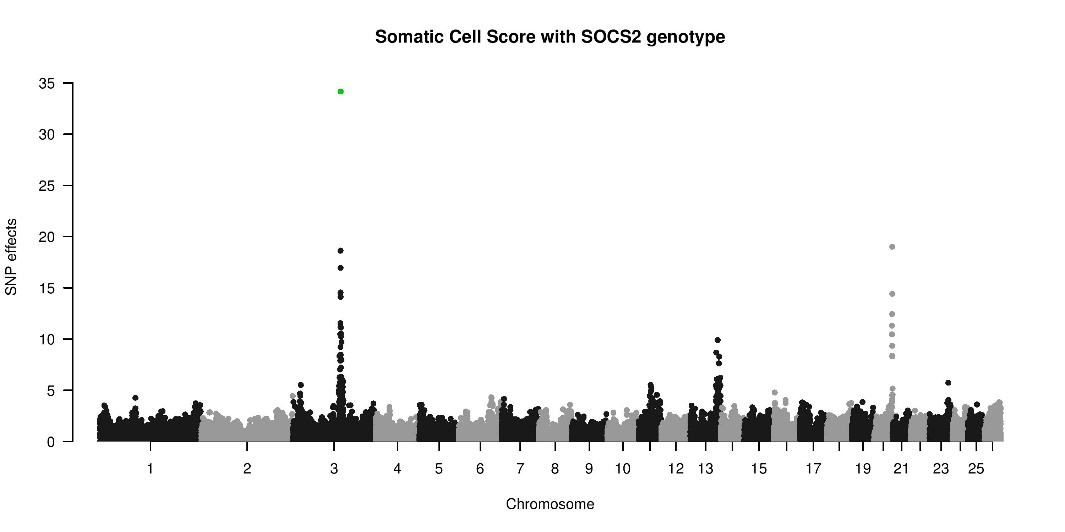


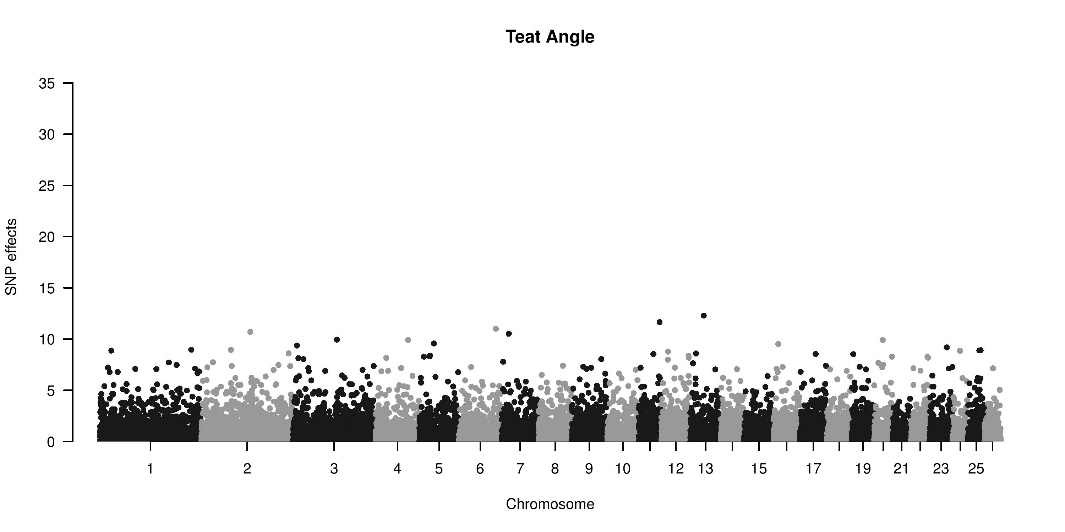

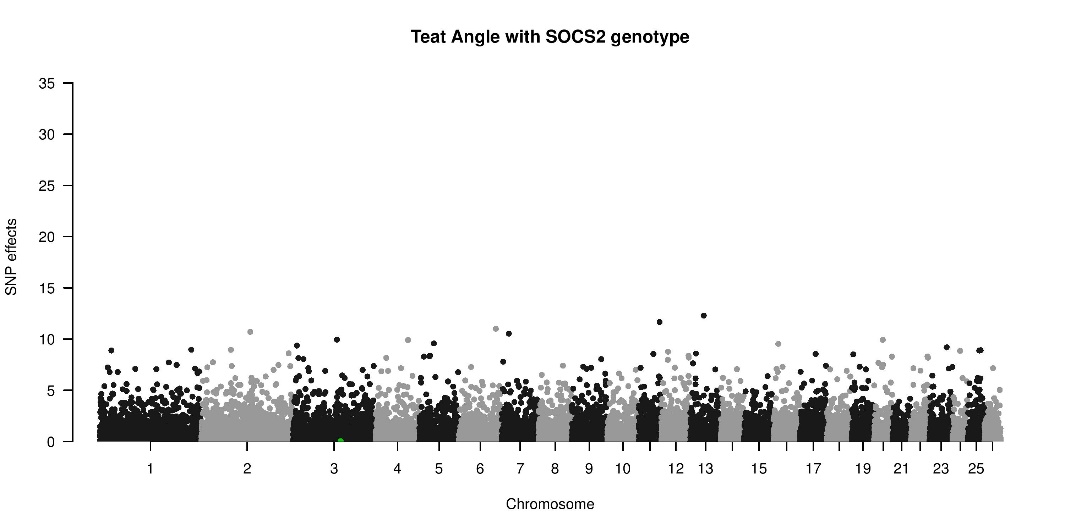

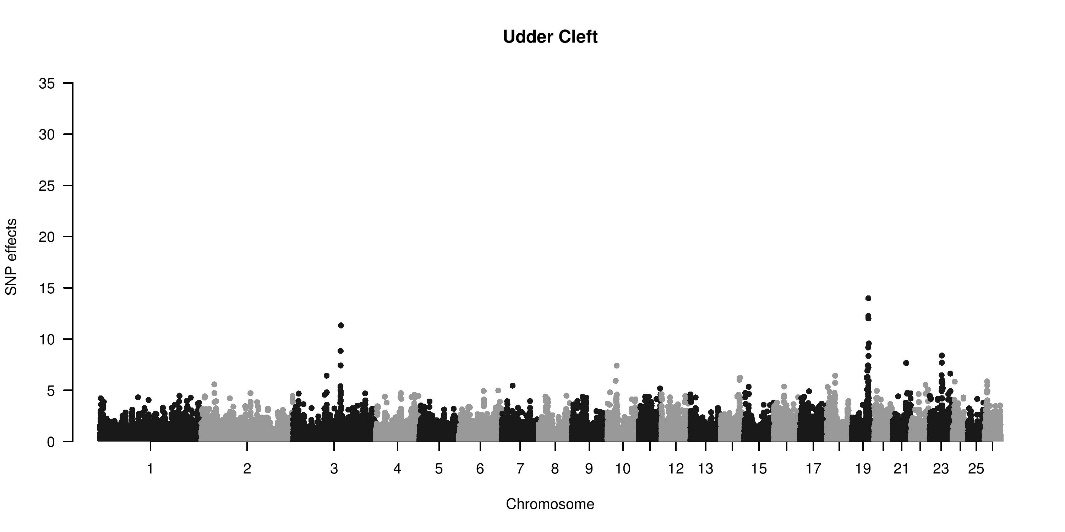

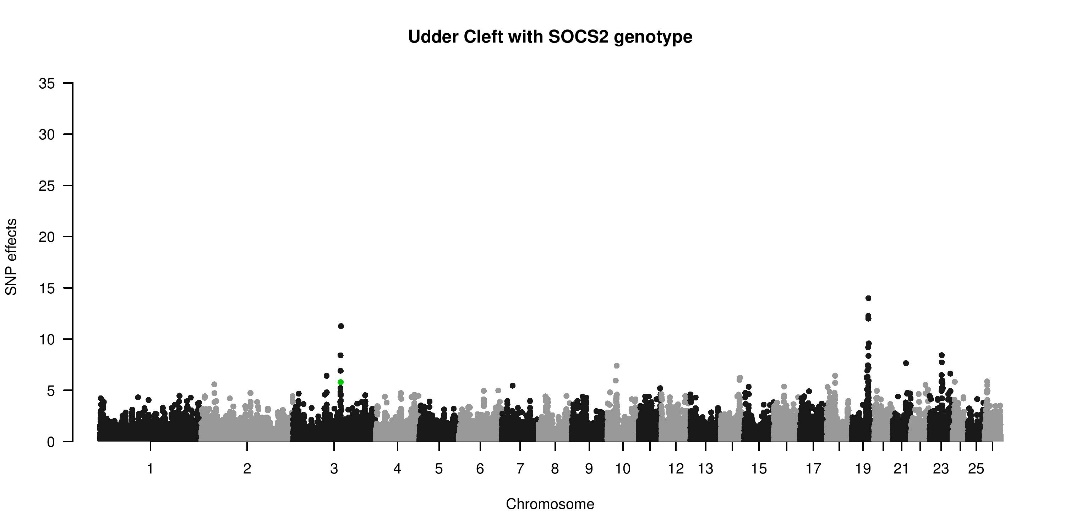

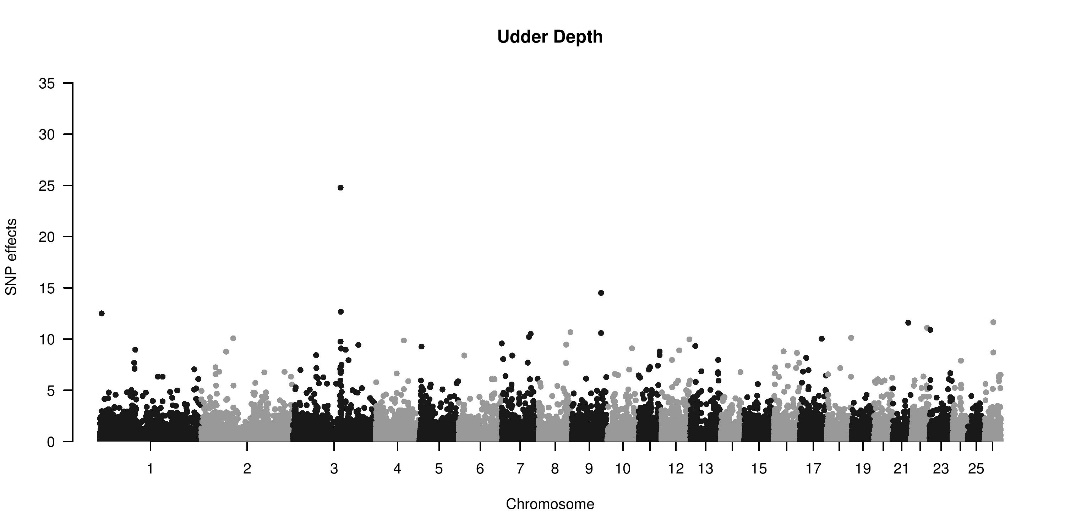

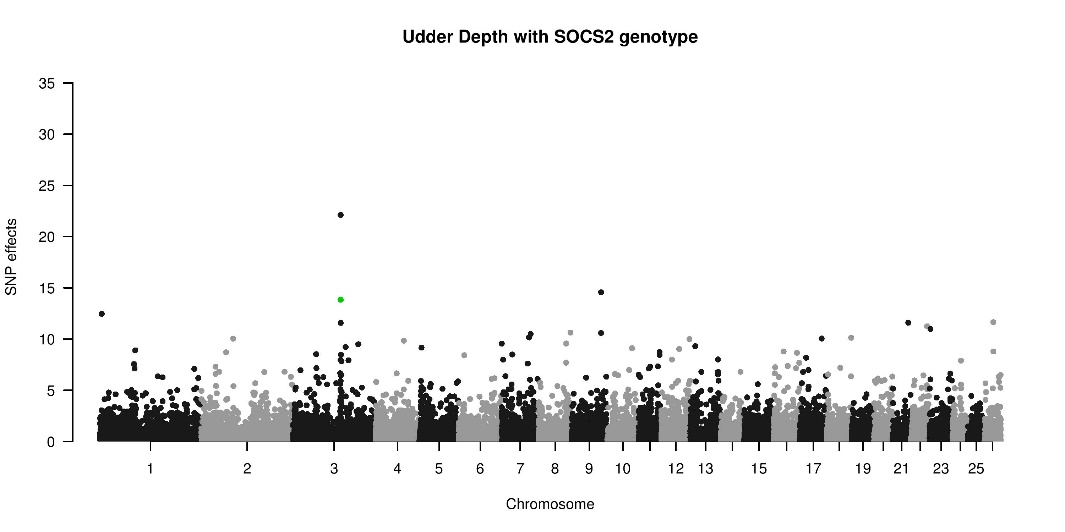


Figure S5 Manhattan plots of estimated variance explained by 20 adjacent SNPs using the best WssGBLUP approach for each phenotype (second iteration). The horizontal red line represents the threshold of 1% adopted in this study. On the left are presented the analyses without the *SOCS2* genotype among the markers and on the right, with the *SOCS2* genotype.


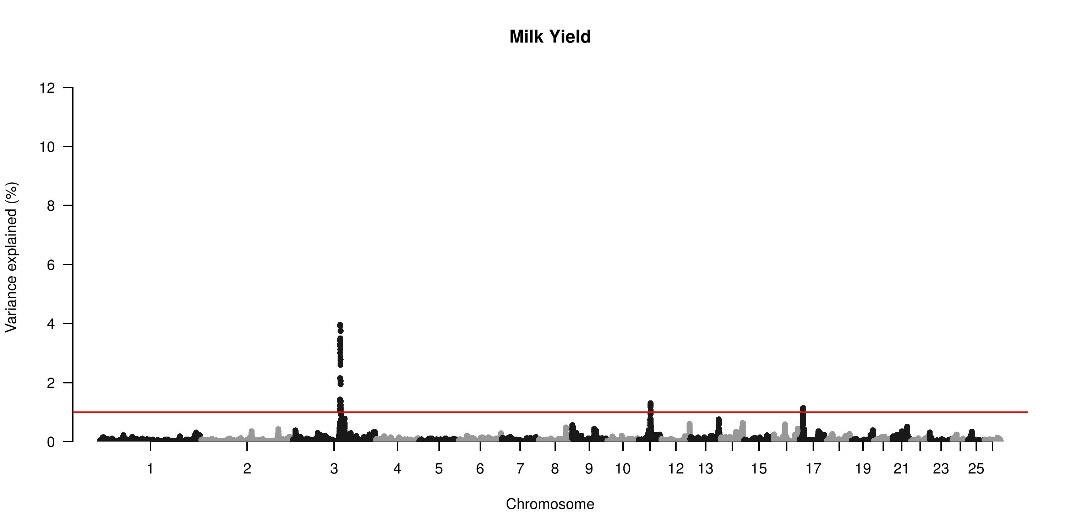

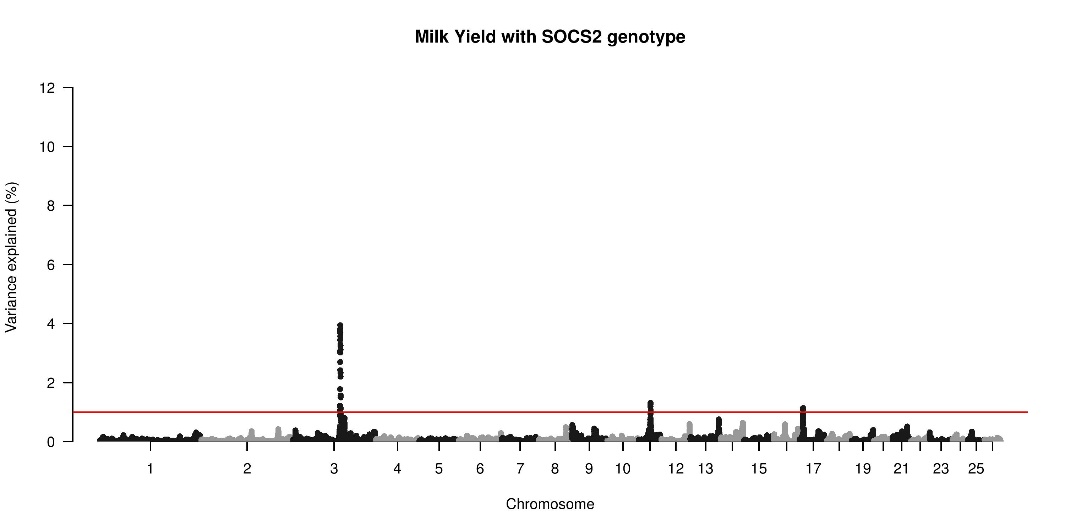

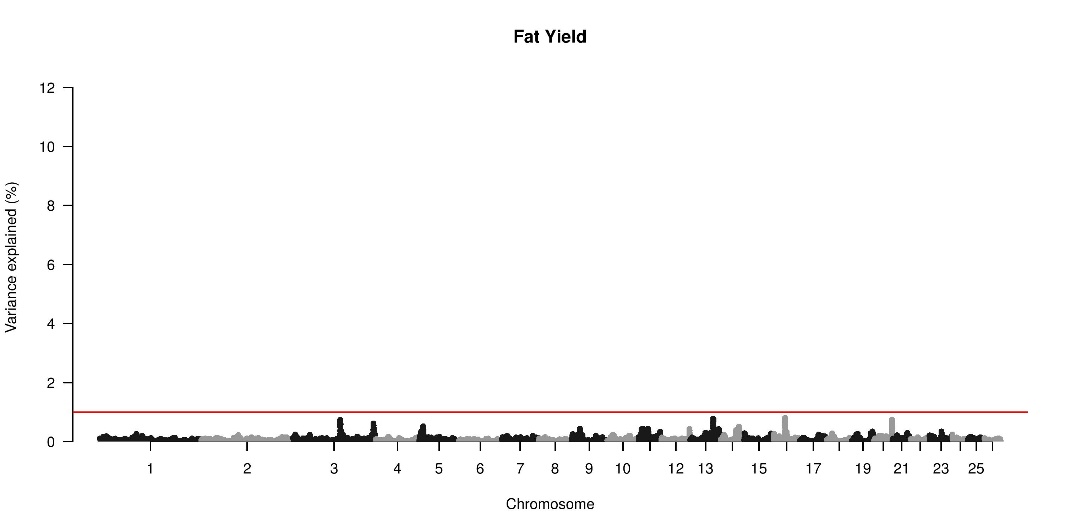

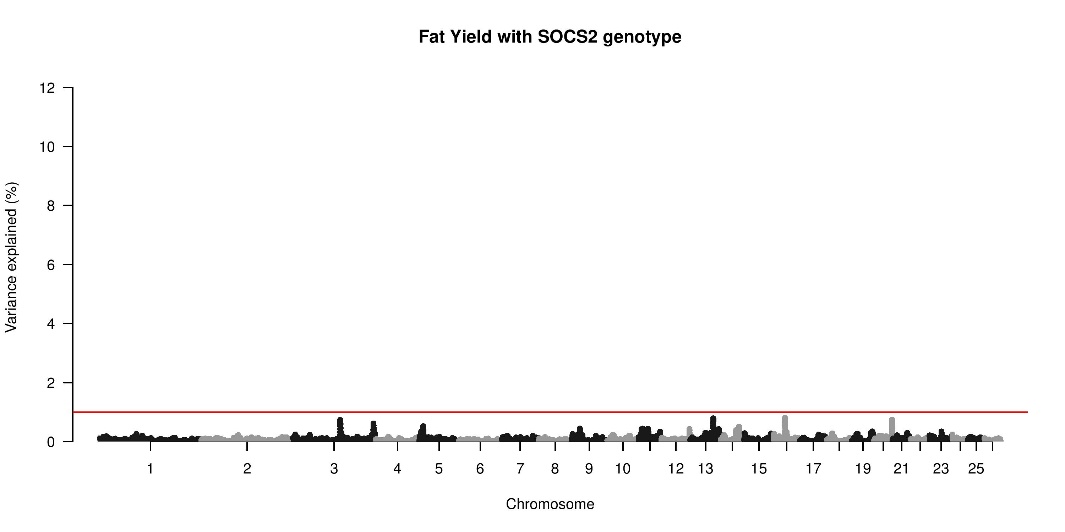

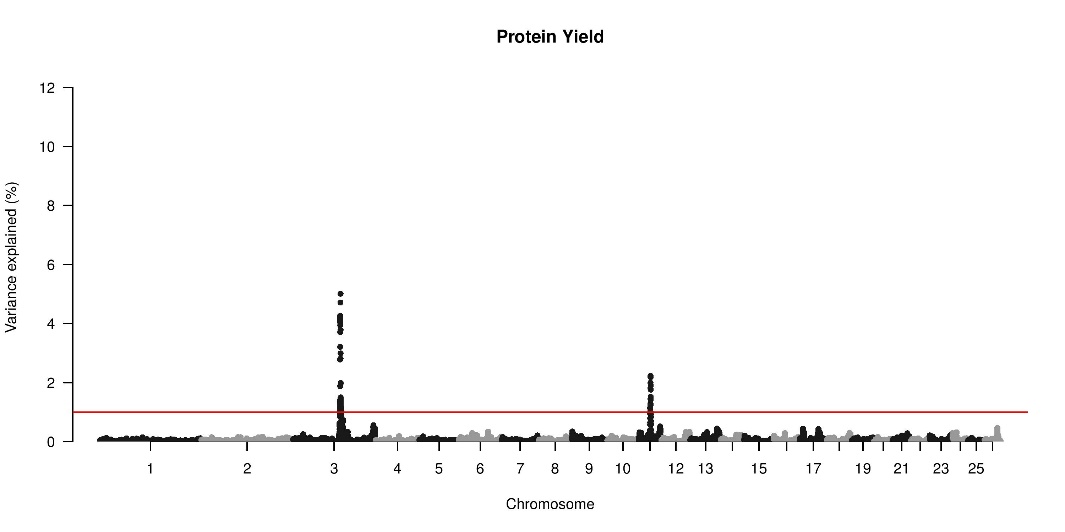

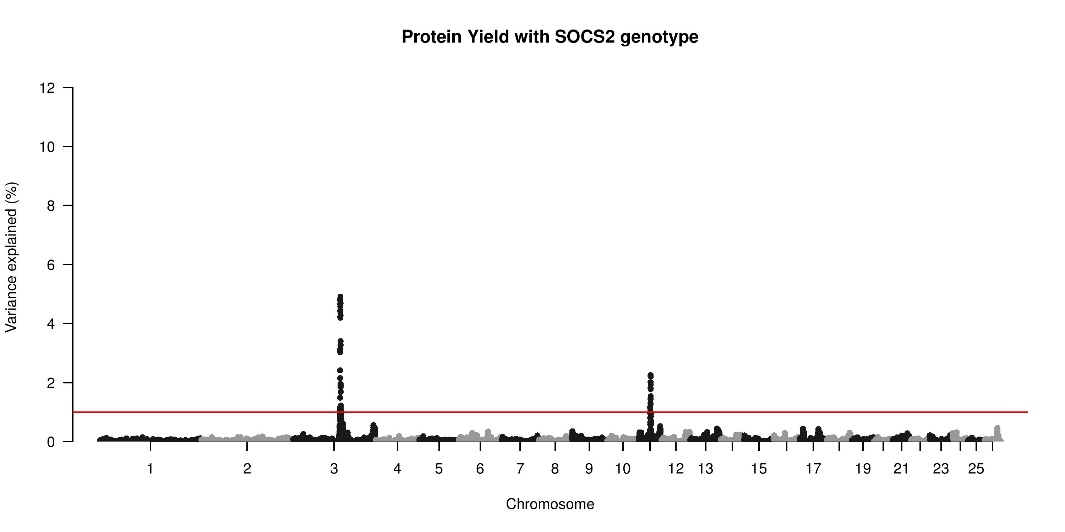

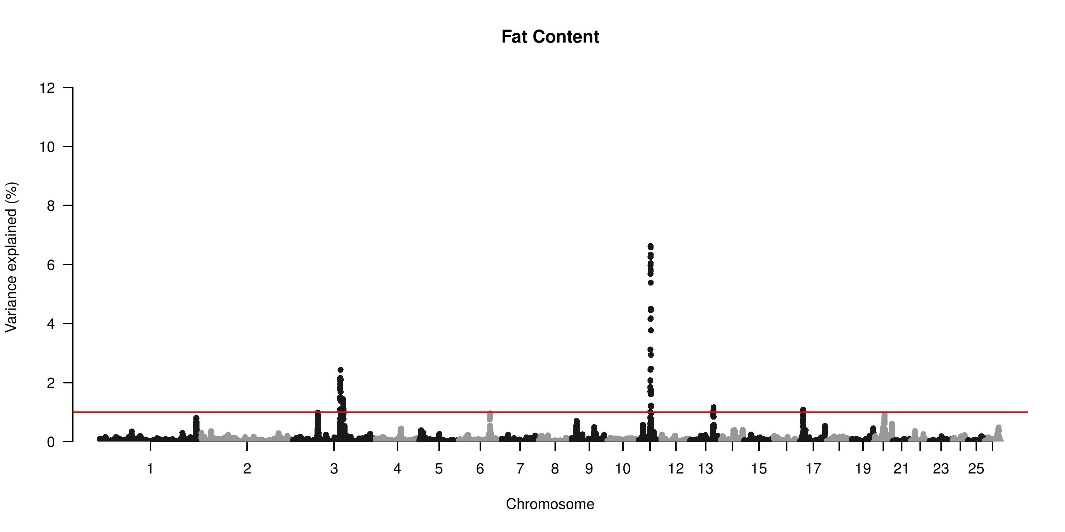

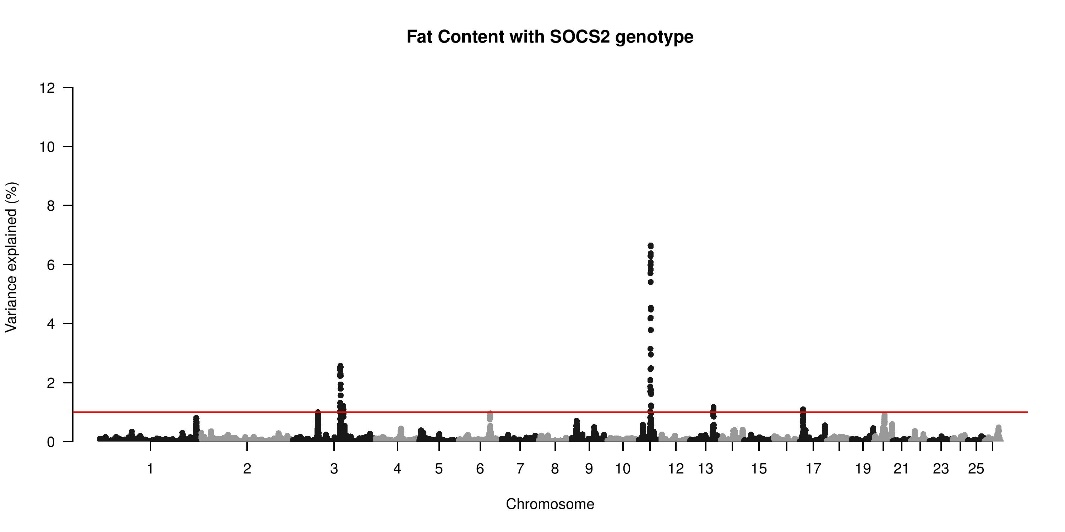

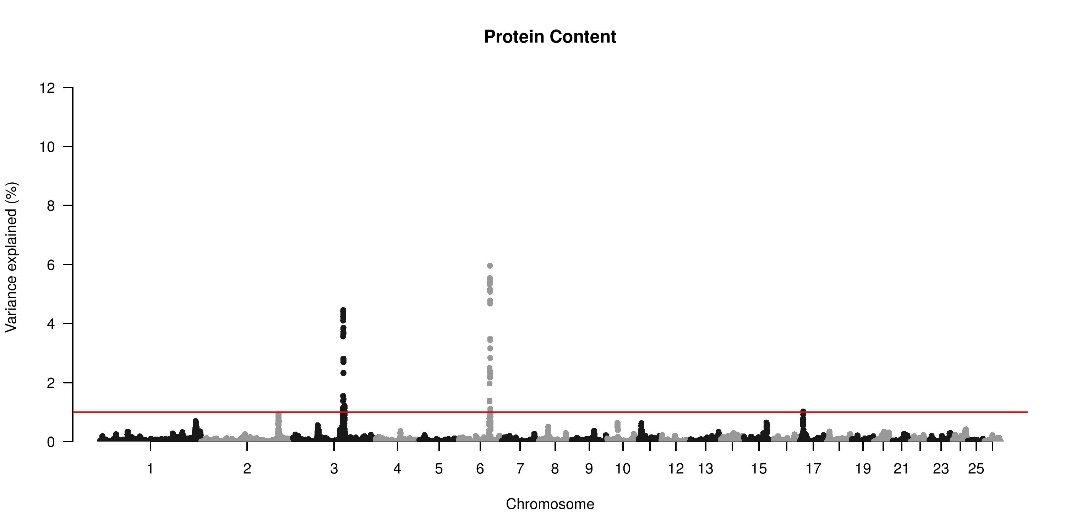

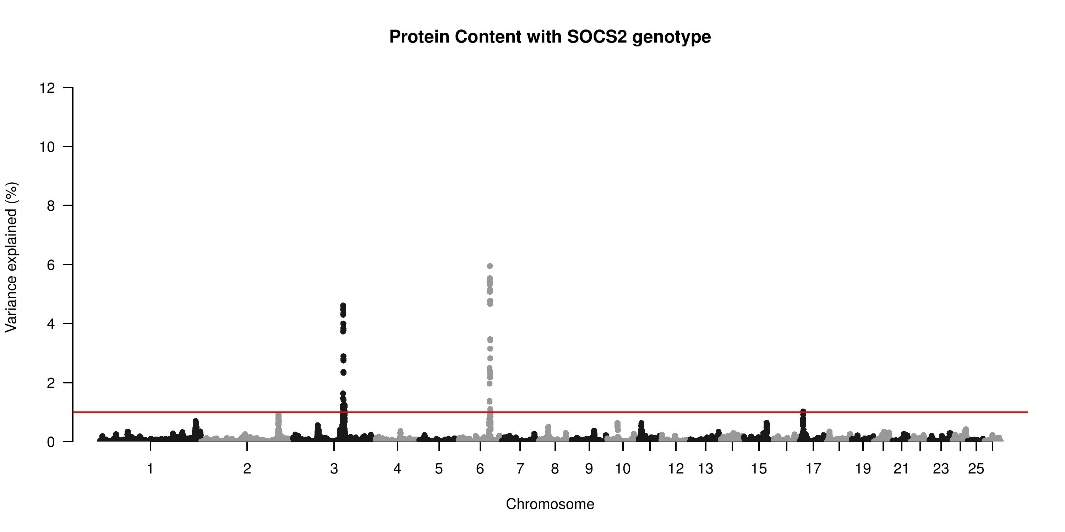


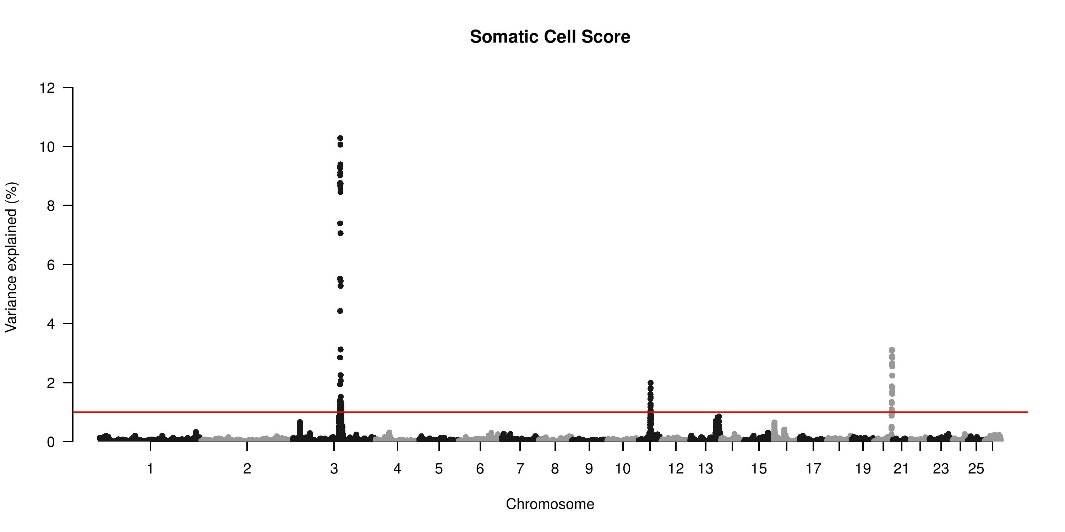

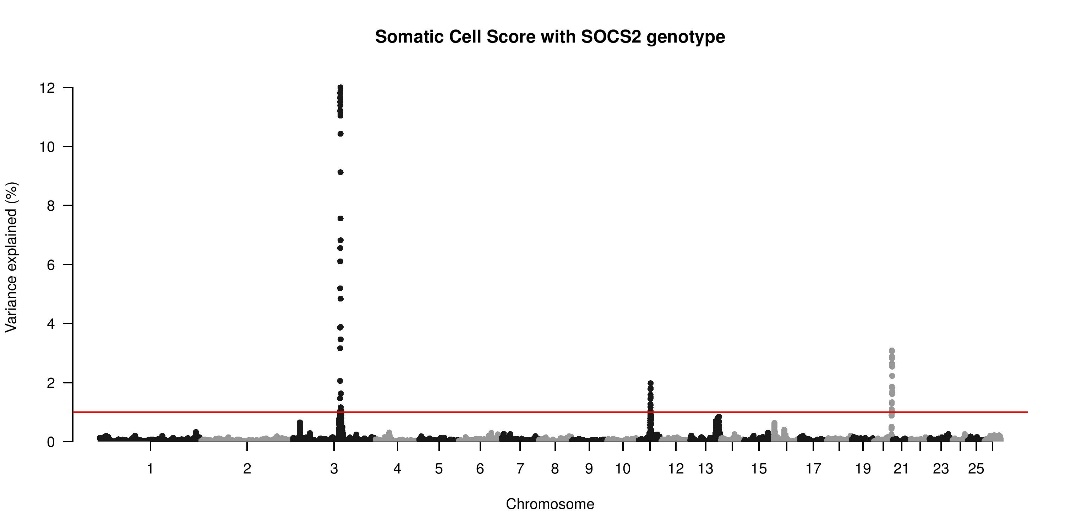


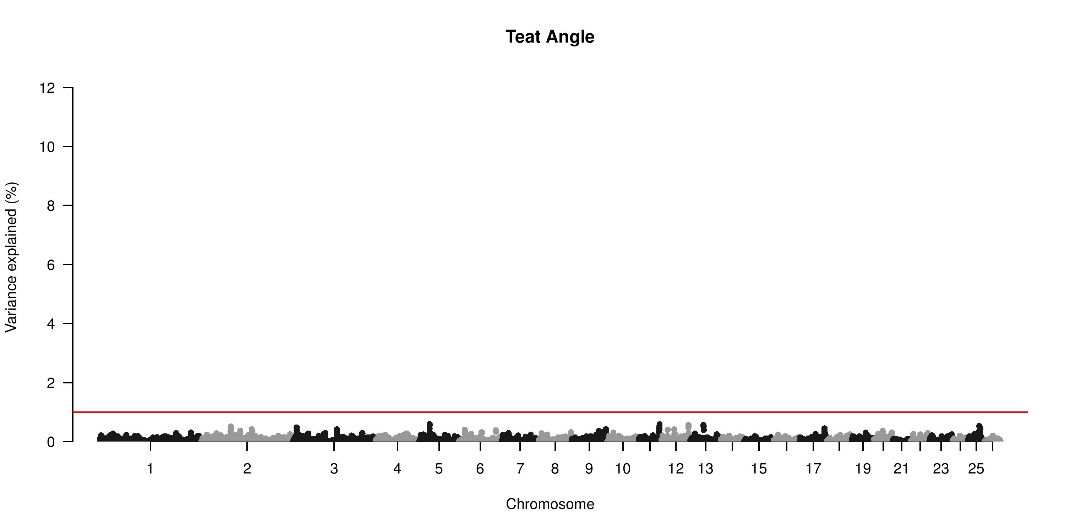

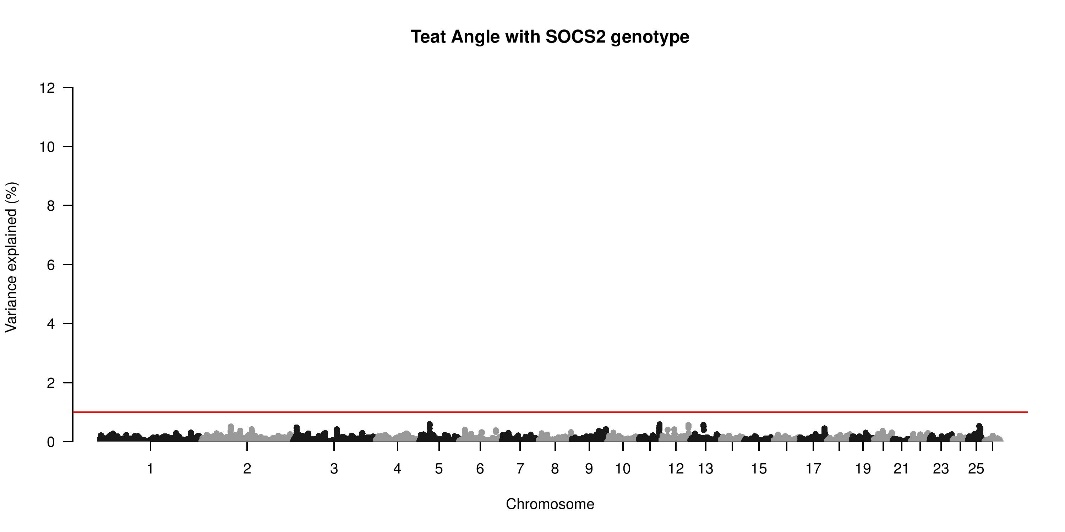

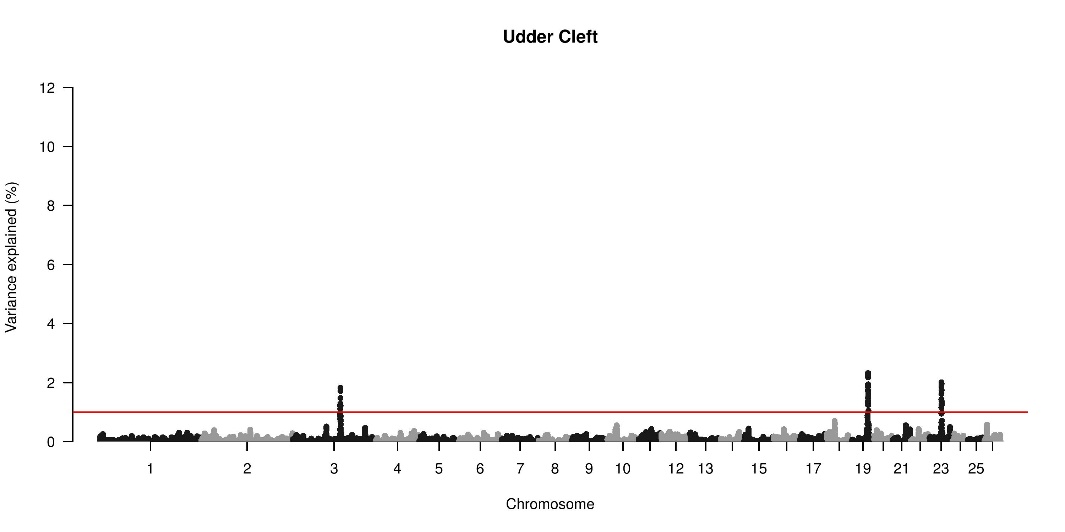

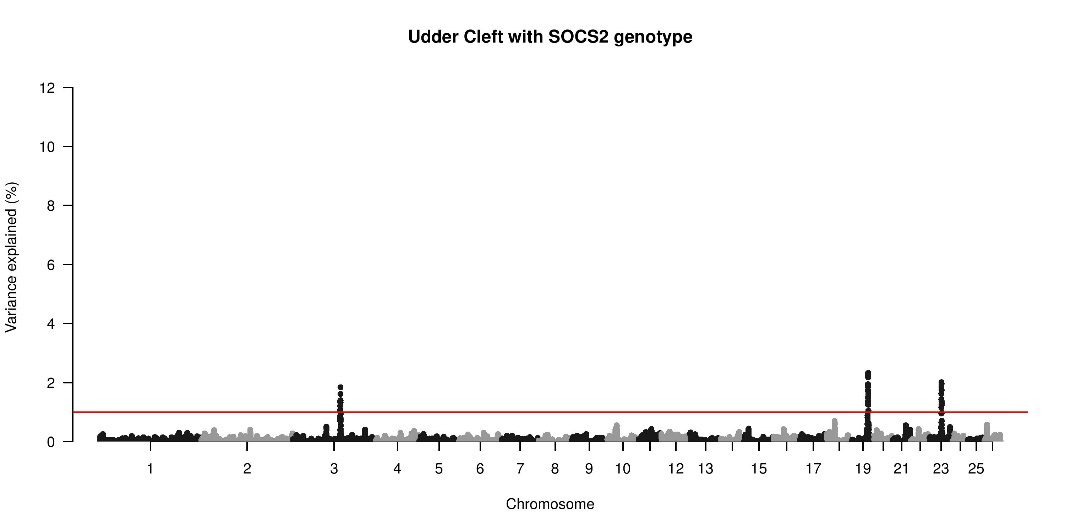

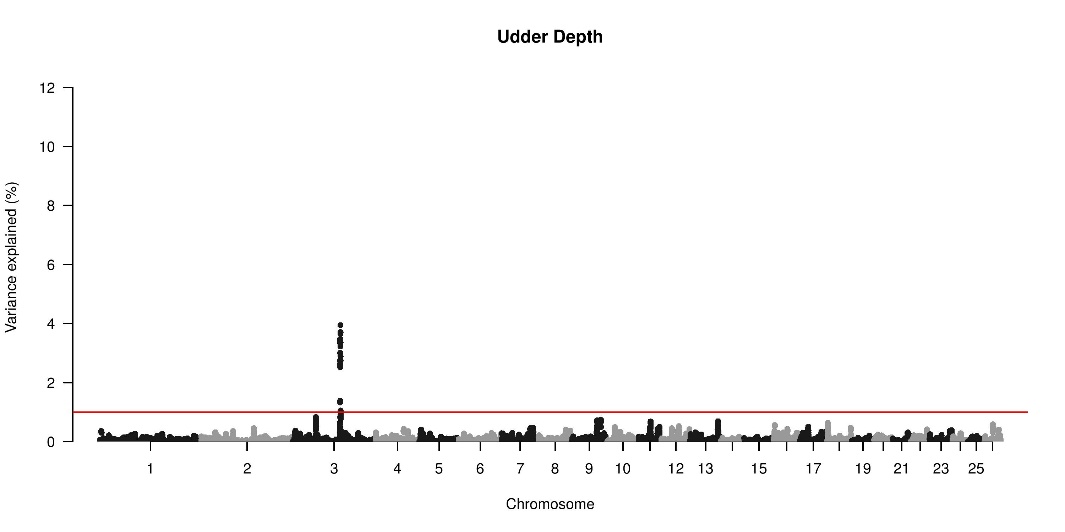

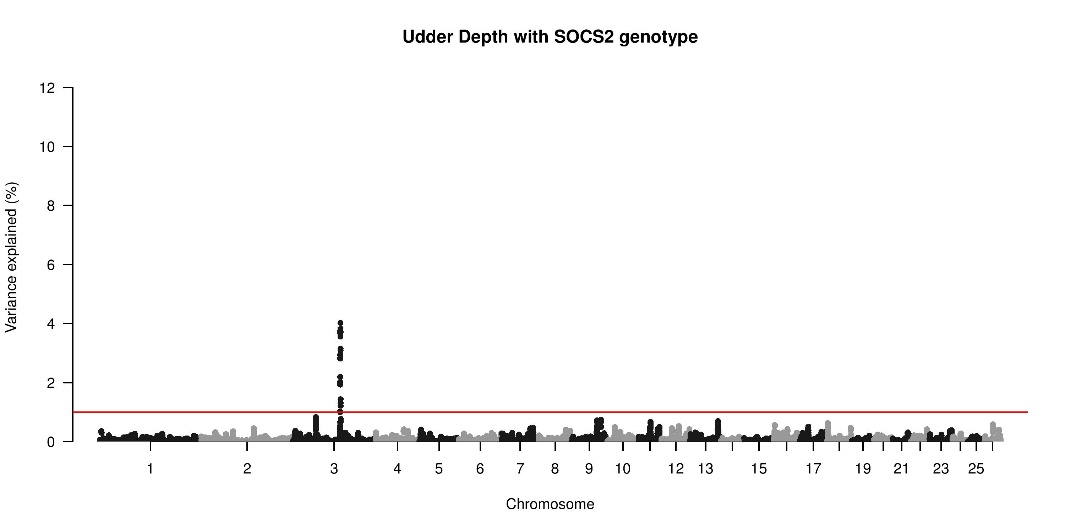

Supplement: Supplementary file 1 — Figure S1 Visualization of linkage disequilibrium (r2 × 100) between the 40 markers closest to the SOCS2 point mutation (rs868996547, in the middle). Figure S2 Visualization of linkage disequilibrium measured as squared correlation coefficient (r2) according to distance between markers on the 50 K ovine SNP chip. Figure S3 Components estimations according to the different models. One-trait methods correspond to eqs. (1) and (2) and two-traits methods to eqs. (3) and (4). Figure S4 Manhattan plots of estimated SNP effects using the best WssGBLUP approach for each phenotype (second iteration). On the left are presented analysis without the SOCS2 genotype among the markers and on the right, with the SOCS2 genotype (green point). Figure S5 Manhattan plots of estimated variance explained by 20 adjacent SNPs using the best WssGBLUP approach for each phenotype (second iteration). The horizontal red line represents the threshold of 1% adopted in this study. On the left are presented the analyses without the SOCS2 genotype among the markers and on the right, with the SOCS2 genotype. (DOCX 2190 kb) [file 12864_2019_6068_MOESM1_ESM.docx]
